# Genome-resolved tracking of *Penicillium commune* in a bakery facility highlights long-term environmental persistence

**DOI:** 10.3389/ffunb.2025.1712444

**Published:** 2025-12-05

**Authors:** Annette Fagerlund, Charlotte Kummen, Anette Wold Åsli, Cathrine Kure Finne

**Affiliations:** Department of Food Safety and Quality, Nofima - Norwegian Institute of Food, Fisheries and Aquaculture Research, Ås, Norway

**Keywords:** *P. fuscoglaucum*, *P. commune*, WGS, food spoilage, mould, potato, lompe, lefse

## Abstract

Mould spoilage is a major challenge in bakery production, yet the sources and persistence of contaminating strains remain poorly understood. We applied whole-genome sequencing (WGS) to 68 isolates from potato-cereal wraps and their production environment in a Norwegian bakery. Barcode-based identification using ITS, *BenA*, *CaM*, and *RPB2* confirmed that 65 isolates belonged to the *Penicillium commune*/*Penicillium fuscoglaucum* lineage but could not fully resolve species status or resolve strain-level differences. Genome-wide comparison using Mash placed these isolates in a single clade within series *Camembertiorum*, distinct from cheese-associated taxa. SNP analysis revealed extremely low diversity within the main cluster (up to 60 SNPs after recombination filtering) and demonstrated that genetically similar strains persisted in the facility for 15 months, spanning multiple products and environmental samples. No consistent association with potato suppliers or production dates was detected, indicating that long-term environmental reservoirs were the main source of contamination. These findings show that persistent clonal lineages can survive routine cleaning in dry bakery environments, enabling recurrent contamination. WGS provided the strain-level resolution needed to uncover this persistence and clarify phylogenetic placement, underscoring its value for monitoring and controlling mould spoilage in food production.

## 1 Introduction

Mould growth is a major cause of food spoilage, leading to economic losses, food waste, and potential health risks from mycotoxins. Bakery products are particularly susceptible, with contamination occurring during production or storage. Previous studies have shown that food processing environments harbor diverse mould species introduced via *e.g.*, raw materials, air, or surfaces (Finne, 2023; Kure et al., 2004; Schirmer et al., 2018). In a previous study, *Penicillium commune* was identified as the primary spoilage mould on potato-cereal soft wraps (lompe or lefse) from a Norwegian producer (Finne, 2023). The same species was frequently detected in the facility’s air and on surfaces in the production facility (Finne, 2023). Potential sources of spore introduction included potatoes carrying soil on the surface, wheat flour, and through air. However, species-level identification of *P. commune* alone was insufficient to elucidate contamination routes or define effective preventive measures. Tracing mould contamination requires insight into both its sources and spread within processing environments. The necessary taxonomic resolution depends on the diversity present, and strain-level differentiation becomes essential when multiple populations of the same species exist and only some cause spoilage. Ultimately, identifying persistent environmental reservoirs is critical for designing targeted interventions and preventing recurrent contamination.

Currently, taxonomic identification of *Penicillium* moulds to species level relies on classical morphological criteria (Samson et al., 2010) and DNA barcodes such as the internal transcribed spacer (ITS) region of the rRNA gene, β-tubulin (*BenA*), calmodulin (*CaM*) and the RNA polymerase II second largest subunit (*RPB2*) (Visagie et al., 2014). GenBank accessions for these four barcodes are now routinely included in formal descriptions of *Penicillium* species (Visagie et al., 2014). However, these markers often lack resolution for closely related species, and genome-scale analyses have revealed frequent misidentifications (Houbraken et al., 2021; Steenwyk et al., 2024; Visagie et al., 2023).

Various molecular typing methods have been used to differentiate strains within fungal species (Rico-Munoz et al., 2019). Multilocus sequence typing (MLST) is frequently used in medical mycology to type moulds. However, in food mycology, the application is limited (Rico-Munoz et al., 2019) and can usually not discriminate below the species level. Amplified fragment length polymorphism (AFLP) was found to be a valuable method to identify cheese-contaminating fungi below species level and suggested spore transfer via staff or equipment, with the same strains repeatedly detected in the processing plant for over a year. However, AFLP lacked resolution to confirm clonal origin (Kure et al., 2003). In contrast, analyses based on whole-genome sequencing (WGS) offers superior resolution and is now standard for tracking bacterial pathogens like *Listeria monocytogenes* and *Salmonella* (Brown et al., 2019). Comparable approaches for spoilage moulds remain largely unexplored, although recent work has used WGS and single nucleotide polymorphism (SNP) analysis to track the origin and dispersal of fungal pathogens, *e.g.*, *Pseudogymnoascus destructans* (Drees et al., 2017) and *Zymoseptoria tritici* (Singh et al., 2021).

The aim of this study was to apply WGS analysis to characterize the diversity and relatedness of *P. commune* isolates associated with spoilage of potato–cereal wraps and their production environment. Specifically, we sought to (i) confirm and refine species identification of isolates from products and environmental sources, (ii) assess the genetic diversity within the dominant species, and (iii) evaluate whether WGS-based phylogenetic analysis can reveal persistence or dissemination patterns of strains within the facility.

## 2 Materials and methods

### 2.1 Source and selection of isolates

A total of 68 isolates were selected from a previous study on spoilage mould on potato-cereal soft wraps and their production environment (Finne, 2023). As described, isolates from products with visible mould spots returned from retail (50 unopened packages, produced from September 2019 to January 2021) were obtained by cultivating small sections of 150 mould colonies on malt extract agar plates (MEA; Oxoid). MEA is a general-purpose mycological medium (acidic pH, high carbohydrate content) that promotes sporulation and morphological differentiation of yeasts and molds used in species-level identification. Furthermore, to identify potential sources of the spoilage mould in the environment, the air and surfaces in the facility were sampled on 15 and 7 days, respectively, across multiple zones within the bakery in October to December 2019 and January, February and April 2020. Air was sampled using passive settle plates (exposure 1 h) containing Dichloran 18% glycerol agar (DG18; Oxoid) with supplements (Samson et al., 2010). DG18 agar is a low water activity medium that includes dichloran to restrict colony spread by mucoraceous fungi, thereby improving recovery of xerotolerant food or indoor fungi in airborne samples (Samson et al., 2010). Surface sampling was performed by swabbing approximately 100 cm^2^ with a sterile cotton swab before the swab was streaked onto MEA plates. The majority of environmental samples were collected from the bakery production area, which contained two parallel production lines and a shared packaging line and physically separated from raw material handling rooms (potato storage, washing, and cooking located on a different floor). Selection of MEA for surfaces paralleled product media to maintain comparability of product-relevant isolates while supporting sporulation and trait expression for morphology-based identification. All agar plates were incubated 7 days at 25 °C. Morphological identification to species level was performed by cultivation on diagnostic media (Samson and Frisvad, 2004; Samson et al., 2010).

Selection of isolates for WGS was designed to capture diversity relevant to spoilage. For product samples, a subset of *P. commune* isolates was chosen to span the entire sampling period and included products made from raw materials supplied by different farms, ensuring temporal and source diversity. For environmental samples, *P. commune* isolates from air and surfaces were selected to represent the full range of sampling dates and facility locations, with most originating from the bakery area where contamination was most critical. Overall, isolates were chosen to represent the dominant spoilage mold across different production days, raw material suppliers, and factory zones, enabling assessment of genetic diversity and persistence. Details of available and selected isolates are provided in **Table 1** and **Supplementary Table S1**. Most selected isolates were phenotypically identified as *P. commune* and/or *P. palitans* (n=66), of which two (MF09503 and MF09504) were further confirmed as *P. commune* or *P. fuscoglaucum* by Sanger sequencing of PCR amplicons generated using ITS primers ITS1/ITS4 (White et al., 1990) and β-tubulin (*BenA*) primers Bt2a/Bt2b (Glass and Donaldson, 1995). In addition, two isolates identified only to the genus *Penicillium* were included.

**Table 1:** Origin of isolates subjected to WGS, with total available isolates in parentheses.

Production or sampling month	Product isolates, by raw material (potato) provider					Environmental isolates	
	Farm A	Farm B	Farm C	Farm D	unknown	Air	Surface
September 2019				1 (3)			
October 2019	1 (1)	1 (2)	3 (7)	1 (8)		0 (5)	1 (1)
November 2019		5 (10)			1 (3)	3^a^ (22)	0 (2)
December 2019						3 (18)	
January 2020	12 (27)					5 (8)	
February 2020						5 (7)	20^b^ (26)
April 2020						0 (21)	0 (9)
June 2020	2 (2)						
October 2020	0 (2)	1 (5)	1 (5)				
December 2020	1 (9)						
January 2021	1 (10)						

a One isolate identified by WGS as *P. rubens*.

b Two isolates initially identified morphologically as *Penicillium* sp., later confirmed by WGS as *P. rubens* and *P. commune*, and a third isolate identified by WGS as *P. polonicum*.

### 2.2 Cultivation, DNA extraction, and sequencing workflow

Fungi were incubated in 10 mL Malt Extract Broth (Oxoid, CM0057) at 25 °C for 5 to 7 days with shaking at 150 rpm. After incubation, the mycelium was harvested, frozen in liquid nitrogen, and ground to a fine powder using a sterile pre-chilled mortar and pestle. Genomic DNA was isolated using the DNEasy Plant Mini Kit (Qiagen). Each sample was eluted in 50 µL Buffer AE and further diluted in 10 mM Tris-HCl pH 8. DNA concentration and purity were assessed using a NanoDrop ND-1000 Spectrophotometer (Thermo Scientific) by absorbance measured at 260 nm and 280 nm. DNA integrity was evaluated by electrophoresis on a 0.7% agarose gel stained with GelRed (Biotium). Libraries were prepared using the Illumina DNA Prep (tagmentation) library preparation kit with 450 bp insert size and sequenced on an Illumina NovaSeq X Plus platform with a 1.5B flow cell and 150-bp PE reads, at the Norwegian Sequencing Centre (http://www.sequencing.uio.no). Sequencing data from this study is available from the NCBI database under BioProject accession number PRJNA1256895.

### 2.3 Genome assembly

Raw reads were filtered on q15 and trimmed of adaptors using fastq-mcf from the ea-utils package (Aronesty, 2011) and then subjected to *de novo* genome assembly using SPAdes v.3.13 (Bankevich et al., 2012) with the careful option and six *k*-mer sizes (21, 33, 55, 77, 99, and 127). Contigs with sizes of <500 bp and *k*-mer coverage of <5 were removed from the assemblies. The average coverage and the quality of the genome assemblies was evaluated using QUAST v5.0.2 (Mikheenko et al., 2018). Sequenced isolates are listed in **Supplementary Table S1**.

### 2.4 Barcode-based species identification

The published GenBank references for the partial β-tubulin (*BenA*), calmodulin (*CaM*), and RNA polymerase II second largest subunit (*RPB2*) gene sequences and the internal transcribed spacer region (ITS) for relevant *Penicillium* species (Houbraken et al., 2020; Visagie et al., 2024) were used as BLAST queries and aligned with the corresponding sequences from target genomes using CLC Main Workbench (Qiagen).

### 2.5 Selection of reference genomes

A total of 560 *Penicillium* spp. genomes available in NCBI Genbank as of 05.06.2025 (including 169 RefSeq references) were downloaded, excluding two MAGs and one assembly identified as contaminated based on its unusually large genome size (68.8 Mb compared to ~35 Mb for *Penicillium*) and the presence of *Aspergillus niger* sequences (GCA_019827475). The genome of *Aspergillus niger* ATCC 1015 was included as an outgroup. In addition, 61 genomes from *Penicillium* series *Camembertiorum* (Ropars et al., 2020) were assembled from raw Illumina reads (BioProject PRJNA655754) using the pipeline described in Section 2.3, except with filtering on coverage <2. Two low-coverage datasets from the study were excluded (SRR12641531 and SRR12641502). The resulting assemblies had N50 values of 118–245 kbp, sizes of 31.8–39.0 Mbp, and 312–1381 contigs (median coverage 32×). In total, 622 reference genomes were included (**Supplementary Table S2**).

### 2.6 Genome-wide similarity analysis using Mash

Pairwise distances were calculated using Mash v2.3 (Ondov et al., 2016) with sketch size 10^5^ and the default *k*-mer size of 21. Neighbor-Joining trees were generated from the Mash output using the BIONJ algorithm (Gascuel, 1997) implemented in the ape package v5.8-1 (Paradis and Schliep, 2019) in R v4.5.0 (R Core Team, 2025) as function bionjs. Trees were visualized using the R package ggtree v3.16.0 (Yu, 2020). Two trees were prepared, the first with all genomes and *Aspergillus niger* ATCC 1015 as outgroup and the second with selected *Penicillium* section *Fasciculata* series *Camembertiorum* genomes and *Penicillium nordicum* UASWS BFE487 as outgroup. Mash topology was used for broad-scale placement and was cross-checked against species-level taxonomy and RefSeq annotations to confirm major clades.

### 2.7 SNP calling and filtering

Reads were mapped to the complete *P. fuscoglaucum* Pf_T2 genome (Luciano-Rosario et al., 2024) (GenBank GCA_040250155.1) using BWA-MEM (Li, 2013), and alignments were processed with Samtools and Picard to add read groups and remove PCR duplicates. Variant calling followed the GATK pipeline (McKenna et al., 2010): HaploTypeCaller with a sample ploidy of 1 was run twice, with base quality recalibration and local realignment, to generate gVCFs per isolate. The minimum base quality was set to 20 and the minimum phred-scaled confidence threshold for variant calling to 30. Repetitive regions were masked using RepeatMasker and RepeatModeler (Flynn et al., 2020; Smit et al., 2015) and multi-nucleotide polymorphisms were removed. The individual files were jointly genotyped (GenomicsDBImport followed by GenotypeGVCFs). Variants were restricted to SNP sites and hard-filtered using site-level thresholds (QD < 2.0, FS > 60.0, MQ < 40.0, SOR > 3.0, MQRankSum < –12.5, ReadPosRankSum < –8.0) and genotype-level filters (GQ < 50, DP < 5, allele balance < 0.9). Sites with missing data or spanning deletions were removed (bcftools view -e ‘GT=“.” || ALT=“*”‘). For the 64−isolate cluster, MF09489 was removed prior to filtering missing data and spanning deletions, and only sites polymorphic in the 64 were retained.

To reduce linkage and recombination effects, linkage disequilibrium (LD) decay was quantified using PLINK v1.9 (Chang et al., 2015) on the two filtered vcf files (with and without MF09489), additionally restricted to biallelic SNPs and MAF>0.02. In both cases, LD decayed to r² ≤ 0.2 at ~20 kb (**Supplementary Figure S1**). SNPs were thinned to one per 20 kb and restricted to biallelic sites. The final dataset was converted to concatenated SNP alignments for phylogenetic analysis, and pairwise distances were calculated using snp-dists.

### 2.8 Maximum likelihood phylogenetic analysis

Evaluation of models for sequence evolution was performed using the advanced model selection option in the ModelFinder program (Kalyaanamoorthy et al., 2017) implemented in the software package IQ-TREE2 v2.1.2 (Minh et al., 2020), with the nucleotide frequency count for the *P. fuscoglaucum* Pf_T2 reference genome input using the -fconst option, applying the reconstituted DNA approach to correct for ascertainment bias (Leaché et al., 2015). The best model selected using the Bayesian information criterion, *i.e.*, HKY+F+I, was used in the downstream Maximum Likelihood (ML) analysis. ML phylogenetic inference was performed on the concatenated SNP alignment (variable positions among the 64 isolates only) including the Pf_T2 reference genome added as a projection onto the polymorphic positions to root the tree, using IQ-TREE2 with 1000 rounds of standard nonparametric bootstrap replicates to generate node supports (-b 1000 option) and incorporating the nucleotide frequency count for the reference genome to correct for ascertainment bias. The tree was visualized using Interactive Tree Of Life (iTOL) v7.2.2 (Letunic and Bork, 2024).

### 2.9 Statistical assessment of clustering

Whether specific potato suppliers or production dates were overrepresented within phylogenetic subclusters was tested. Subclusters were defined as internal nodes with bootstrap support ≥95 and containing 5 to 30 isolates in the ML tree. A sensitivity analysis was also performed at a relaxed cutoff of ≥85%. For each subcluster, supplier enrichment among product isolates was assessed using Fisher’s exact test against the overall distribution of suppliers, with Benjamini–Hochberg FDR correction. Temporal clustering was evaluated by comparing the observed median pairwise date difference within each subcluster to an empirical null distribution generated from 2000 random permutations of production/sampling dates across all isolates. The empirical p−value was the fraction of permutations with a median ≤ the observed, followed by FDR correction. Analyses were implemented in Python using numpy and scipy.

## 3 Results and discussion

### 3.1 Overview of isolates selected for WGS

In a previous study (Finne, 2023), *P. commune* was identified as the primary spoilage mould on potato-cereal soft wrap products from a Norwegian producer. Of 112 isolates recovered from 50 unopened packages with visibly mouldy products (festlefse, lefse, lefserull, lompe, pizzabunn, spelt, speltlompe) collected between October 2019 and January 2021 (15 months), 84% (n=94) were identified as *P. commune* and/or *P. palitans* using traditional morphological methods (cultivation). Two tested isolates were subsequently identified as *P. commune* or *P. fuscoglaucum* using barcode sequencing analysis. From these product isolates, 31 isolates were selected for WGS (**Table 1**).

During approximately the same time period (October 2019 to April 2020) environmental sampling targeted potential sources of the product spoilage mould in the production areas. The primary aim was detection of the spoilage mold associated with product contamination, rather than characterizing the full airborne or surface mycobiota of the facility. In total, 207 environmental samples were collected, and mould was detected in 105 (73%) air samples and 34 (53%) surface samples (Finne, 2023). From these, 16 air and 19 surface isolates identified morphologically as *P. commune* and/or *P. palitans* were selected for WGS. Two additional surface samples identified only to the genus *Penicillium* were also included. All but one of the selected environmental isolates originated from the bakery production area; the single exception (MF09442) was from the potato washing/cooking room located on a different floor. The selected isolates reflect the dominant spoilage mold across time, raw-material sources, and facility locations, rather than a comprehensive survey of all fungi present (**Table 1**).

### 3.2 Classification using barcode sequences analysis

The ITS, *CaM*, *RPB2*, and *BenA* barcode sequences were identical across 65 of the 68 sequenced isolates. These sequences were aligned with all barcodes from *Penicillium* section *Fasciculata* series *Camembertiorum* genomes (**Supplementary Table S3**). For the ITS region, sequences matched reference sequences from *P. biforme*, *P. caseifulvum*, *P*. *commune*, *P*. *fuscoglaucum*, and *P*. *palitans*. For the partial *CaM* gene, sequences were identical to the reference sequences from *P. biforme*, *P. caseifulvum*, *P*. *commune*, and *P*. *palitans*, and showed 1 SNP difference towards the *P. fuscoglaucum* and *P. camemberti* barcodes. The partial *RPB2* gene showed 1 SNP difference towards references from *P*. *camemberti*, *P. caseifulvum*, *P*. *commune*, and *P*. *fuscoglaucum*.

The partial *BenA* sequence, encoding β-tubulin, was identical to *P. commune* and *P. fuscoglaucum* reference sequences, except for a homopolymeric thymidine (poly-T) tract located near the start of the barcode region. This tract consisted of 14 and 16 consecutive thymidines in the *P. commune* and *P. fuscoglaucum* references, respectively (Genbank MN969377 and OR206420), whereas the 65 genome assemblies obtained in this study contained 13 consecutive thymidines. The sequences were also identical to the *P. camemberti BenA* reference sequence (FJ930956), which does not cover this region. A few SNP differences were seen relative to the *P. biforme*, *P*. *palitans*, and *P. caseifulvum* references in regions outside the poly-T tract. Thus, the only barcode marker region distinguishing the 65 isolates from *P. commune* and *P. fuscoglaucum* was the length of this homopolymeric tract. Notably, the closed *P. fuscoglaucum* Pf_T2 genome (GenBank GCA_040250155), generated using Oxford Nanopore Technologies sequencing technology (Luciano-Rosario et al., 2024), contained only 10 thymidines at this site. These findings align with previous reports that *BenA* is the most informative barcode for *Penicillium* species, but also with reports documenting intraspecific variation in this marker (Visagie et al., 2014).

For the two isolates subjected to Sanger sequencing of the *BenA* region (Finne, 2023), chromatograms showed clear signals up to the homopolymeric region, followed by overlapping peaks (**Supplementary Figure S2**). This pattern reflects a mixture of amplicons containing 12 and 13 thymidines, a classic outcome of polymerase slippage during PCR amplification of homopolymeric regions (Shinde et al., 2003). Illumina-based genome assemblies for these isolates consistently showed 13 thymidines, confirming that the heterogeneity originated during PCR rather than representing true genomic variation.

The poly-T tract is located within an intron of the *BenA* gene, so length variation does not affect the reading frame or protein function. However, its presence poses two challenges for using *BenA* as a barcode marker: (i) it introduces sequencing ambiguity due to PCR slippage, and (ii) it represents a genuine source of intraspecific variation, complicating species assignment. To minimize amplification artifacts, the use of high-fidelity polymerases with proofreading activity for PCR is recommended when sequencing *BenA* for taxonomic purposes.

For the remaining three genomes sequenced in the current study, two isolates initially identified as *P. palitans* based on morphology were reassigned based on *BenA* barcode comparison to *P. polonicum* (MF09485) and *P. rubens* (MF09444). The last isolate, previously identified only to the genus level, was classified as *P. rubens* (MF09476) (**Table 1**). These three isolates are not discussed further in this study. Their misidentification – along with the initial classification of several of the other selected isolates as *P. palitans* – underscores the limitations of morphology-based identification of moulds, which is often complicated by overlapping phenotypic traits and the risk of contamination by airborne spores.

### 3.3 Genome assembly metrics

N50 values for the 65 related genome assemblies ranged from 274 to 533 kbp, indicating a high level of contiguity. The total lengths of the assemblies were between 36.0 and 36.6 Mbp, with the number of contigs ranging from 171 to 420. Average coverage depth ranged from 71× to 179×. When the closed reference genome of *P. fuscoglaucum* Pf_T2 (35.1 Mb, Luciano-Rosario et al., 2024) was used as reference in the QUAST evaluation, the genome fractions covered were 92.9% to 93.8% and the percentages of the assemblies that aligned to the reference genome (reference mapped) were 88.9% to 94.0%.

### 3.4 The isolates comprised a single clade

**Figure 1:**
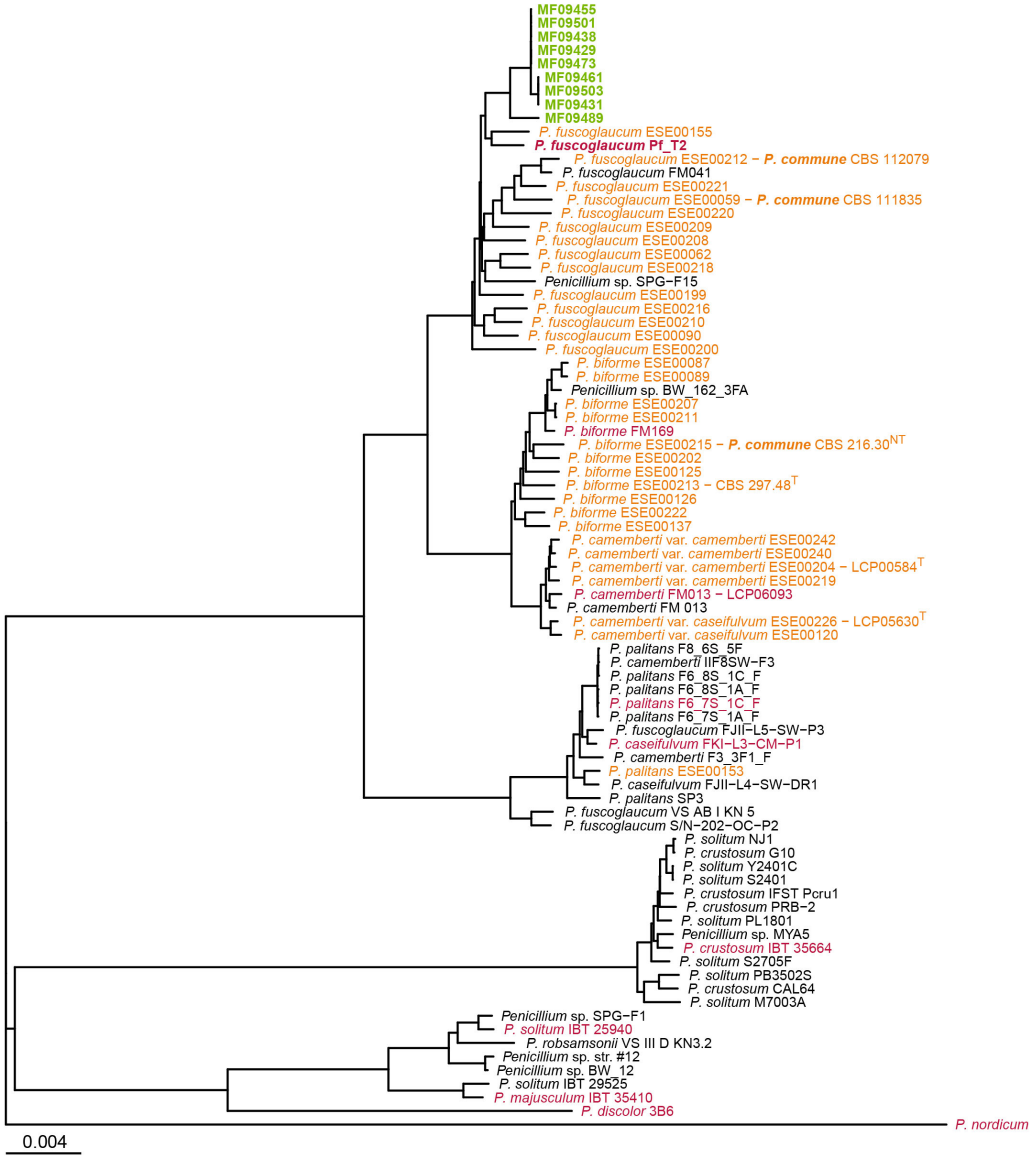
Phylogenetic placement of bakery isolates within *Penicillium* section *Fasciculata*, series *Camembertiorum*. The tree was constructed using the Neighbor-Joining algorithm from pairwise Mash distances. Isolates from the current study are shown in green, genomes from Ropars et al. (2020) in orange, RefSeq reference genomes in red, and other GenBank genomes in black. *P. nordicum* UASWS BFE487 served as the outgroup. Branch lengths are proportional to Mash distance, as indicated by the scale bar. For clarity, clades with many highly similar genomes were downsampled.The full tree including all references is provided in **Supplementary Figure S3**.

Mash analysis (Ondov et al., 2016) of the 68 bakery-associated isolates, alongside 560 publicly available *Penicillium* genomes from NCBI GenBank and 61 genomes from Ropars et al. (2020) was used for broad-scale phylogenetic placement. The analysis revealed that all 65 related isolates formed a single monophyletic clade (**Supplementary Figure S3**; see also **Figure 1**). This tight clustering suggests a highly related population within the bakery environment. One isolate, MF09489, collected from a surface sample in February 2020, was notably divergent from the rest. Mash distances between MF09489 and the other bakery isolates ranged from 2.6×10^3^ to 3.0×10^3^, whereas distances among the remaining 64 isolates were much lower (9.5×10^6^ to 4.4×10^4^), indicating very high genetic similarity.

The closest GenBank reference genomes to the bakery cluster were annotated as *P. fuscoglaucum* Pf_T2 (an indoor air isolate) (Luciano-Rosario et al., 2024), *Penicillium* sp. SPG-F15 (from marine sediment) (Sobol et al., 2019), and *P. fuscoglaucum* FM041 (Ropars et al., 2015). These reference genomes were separated from the bakery cluster by Mash distances of 4.4×10^3^ to 7.8×10^3^. Notably, none of the reference genomes were designated as *P. commune*.

### 3.5 Phylogenetic placement within series *Camembertiorum*

The monophyletic clade containing the 65 bakery isolates was located within the *P. fuscoglaucum*/*P. biforme* lineage, clustering closest to *P. fuscoglaucum* references (Pf_T2 and FM041) (**Supplementary Figure S3**). A condensed Mash-based tree including representative genomes from *Penicillium* section *Fasciculata* series *Camembertiorum* is shown in **Figure 1**. The bakery isolates did not group with the domesticated cheese lineage (*P. camemberti/P. caseifulvum*) or with *P. palitans*, *P. crustosum*, *P. discolor*, or *P. solitum*, indicating that the spoilage population is genetically distinct from cheese-associated lineages.

The ambiguous designation of the 65 isolates as *P. commune* versus *P. fuscoglaucum* reflects the unresolved taxonomy of series *Camembertiorum* (Visagie et al., 2024). The phylogenetic analysis (**Figure 1**) recapitulates the earlier finding that the *P. commune* neotype (CBS216.30, LCP05531) isolated from leaf litter, clusters with the *P. biforme* type strain (CBS297.48) from French cheese (Giraud et al., 2010; Ropars et al., 2020). Ropars et al. (2020) further showed that multiple strains historically labelled *P. commune* instead fall with *P. biforme* or *P. fuscoglaucum*, leading them to argue against continued use of *P. commune*. In contrast, Visagie et al. (2024) retain *P. commune* as a valid species, and only tentatively accept *P. fuscoglaucum*. Further, the valid list for series *Camembertiorum* comprises 11 species; *P. biforme*, *P. camemberti*, *P. caseifulvum*, *P. cavernicola*, *P. commune*, *P. crustosum*, *P. discolor*, *P. echinulatum*, *P. palitans*, *P. solitum*, and *P.* sp*eluncae*, but omits *P. fuscoglaucum* (Houbraken et al., 2020). In our reference genome set, no genomes were annotated as *P. commune*, whereas several were annotated as *P. fuscoglaucum*, including three that clustered within the *P. palitans* lineage (FJII-L5-SW-P3, S/N-202-OC-P2, and VS AB I KN 5). This reflects the frequent misidentified or inconsistently annotated entries found in public genome repositories (Houbraken et al., 2021; Steenwyk et al., 2024; Visagie et al., 2023).

From a nomenclatural perspective, if genomic divergence between *P. commune* and *P. fuscoglaucum* proves minimal and no consistent ecological or phenotypic differences can be demonstrated, the principle of priority under *the International Code of Nomenclature* (Turland et al., 2018) would favour treating them as synonyms, with *P. commune* (Thom, 1910), as the earliest valid name, taking precedence over *P. fuscoglaucum* (Biourge, 1923). Resolving this issue will likely require genome sequence based classification, an approach suggested by several authors (Li et al., 2021; Menicucci et al., 2025; Xu, 2020). However, it should be noted that reclassification of species or other taxa based on WGS data alone is not without controversy, largely due to differing species concepts and concerns about over-splitting or instability in nomenclature (Menicucci et al., 2025; Stengel et al., 2022; Xu, 2020). In the meantime we will use the designation *P. commune* for the bakery population, consistent with our earlier publication (Finne, 2023), while clearly documenting its phylogenetic placement adjacent to isolates currently annotated as *P. fuscoglaucum/P. biforme*.

### 3.6 SNP analysis of *P. commune* genomes

SNP analysis of the 65 *P. commune* genomes, using the *P. fuscoglaucum* Pf_T2 reference (Luciano-Rosario et al., 2024), initially identified 128 thousand SNP sites after strain-level filtering. Removing positions with missing data across any genome reduced this to 69 thousand SNPs. Linkage disequilibrium (LD) analysis showed strong short-range linkage among SNPs, with r² values declining to <0.2 at ~20 kb (**Supplementary Figure S1**). Based on this, LD-guided recombination filtering was applied (one SNP per 20 kb), reducing the dataset to 1264 SNP sites.

Isolate MF09489 differed substantially from the remaining 64 analysed genomes, with 42 thousand SNPs (range 42050 to 42084). Accounting for recombination, this number was reduced to between 570 and 578 SNPs. In contrast, pairwise distances among the remaining 64 genomes were very low: 3 to 92 SNPs (median 35), or 0 to 60 SNPs after recombination filtering. Across these 64 closely related isolates, the total number of variable sites was 344 SNPs before and 208 SNPs after recombination filtering.

These results strongly support that the population represents a clonal expansion within the bakery environment, with negligible divergence among isolates. The extremely low SNP distances among the 64 isolates align with patterns observed for clonal populations reported in other fungi. For instance, intra-host diversity in *Candida glabrata* can reach up to 96 SNPs and still be considered clonal (Misas et al., 2024), while global populations of *Penicillium digitatum* show only ~0.06 SNPs/kb, indicating very limited variation within lineages (Julca et al., 2015). In our dataset, 208 variable positions across a ~36 Mb genome represent negligible divergence, suggesting that all isolates belong to a single clonal lineage.

MF09489 differed from the *P. fuscoglaucum* Pf_T2 reference by about 57 thousand SNPs (1035 SNPs after recombination filtering). In comparison, the remaining 64 genomes showed approximately 38 thousand SNP difference (range 38311 to 38346) relative to the same reference (between 807 and 815 SNPs after recombination filtering). Thus, the 64 genomes were genetically closer to *P. fuscoglaucum* Pf_T2 than to MF09489, whereas MF09489 was closer to the 64-genome cluster than to Pf_T2. These results contrast with the Mash analysis, which suggested that MF09489 was more closely related to the other 64 isolates than to *P. fuscoglaucum* Pf_T2.

### 3.7 Phylogenetic analysis reveals a persistent clonal lineage

**Figure 2:**
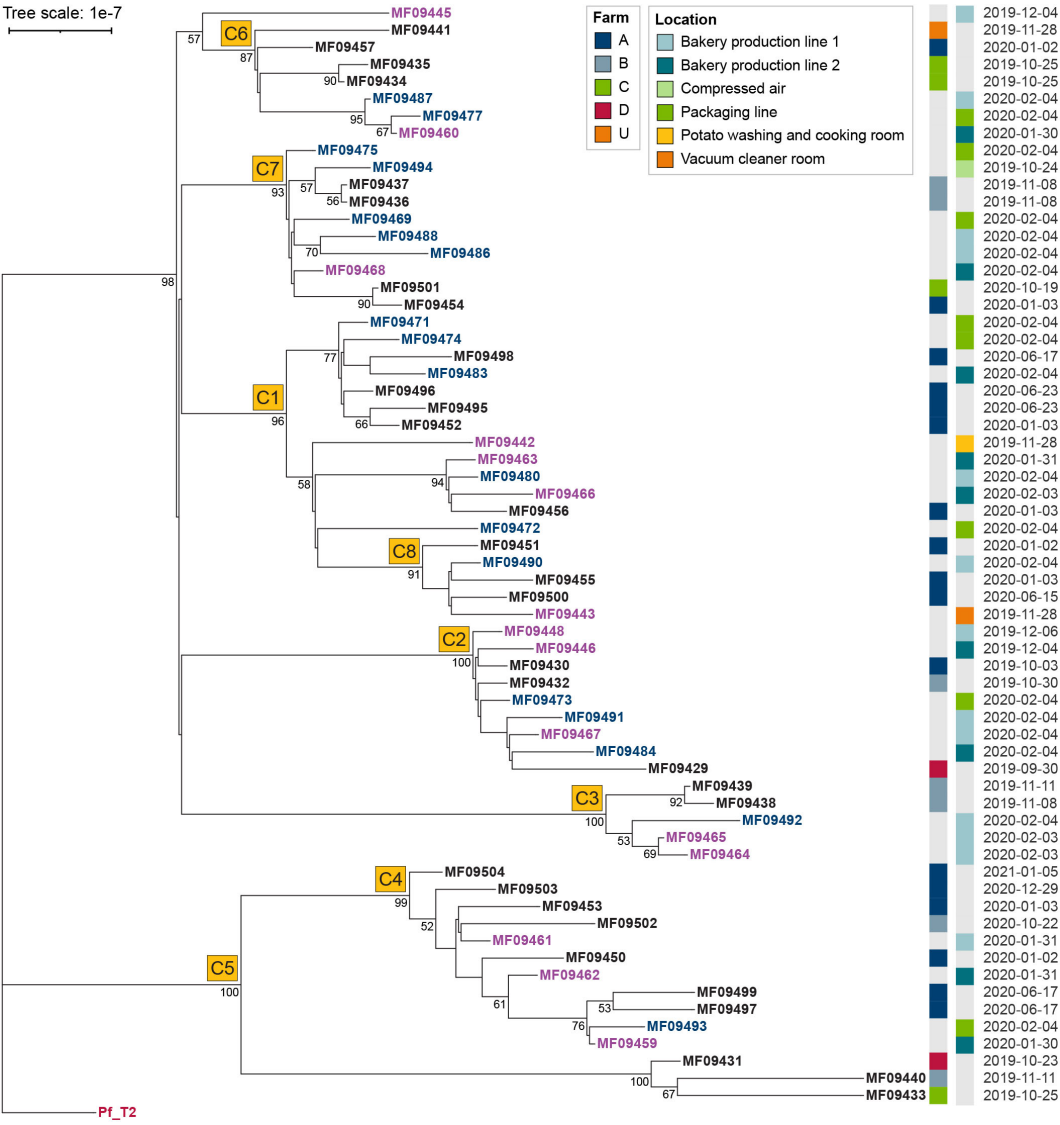
Maximum Likelihood phylogeny of 64 *P. commune* isolates, based on 208 SNP positions remaining after recombination filtering. The tree is rooted on the Pf_T2 reference genome, which was included in the SNP alignment as a projection onto positions polymorphic among the 64 isolates; invariant sites were excluded. The tree was inferred using IQ-TREE under the HKY+F+I model with 1000 bootstrap replicates; branch labels indicate bootstrap support values >50. Branch lengths are proportional to substitutions per site (scale bar). Node labels are colored by sample type: product (black), air samples (purple), surfaces in bakery (blue). For product isolates, the potato supplier (anonymized as Farm A, Farm B, etc.) and production date are shown; for environmental isolates, sampling location and date are indicated. Subclusters identified for statistical assessment of supplier enrichment and temporal clustering (see **Supplementary Table S4**) are marked with labels C1–C8 next to the corresponding nodes.

Maximum Likelihood phylogenetic analysis of the 64 nearly identical *P. commune* genomes revealed several subclusters containing isolates from both products and environmental samples collected months apart (**Figure 2**). These subclusters likely represent minor genetic differences that accumulated over time within a persistent lineage or arose from sampling closely related subpopulations, rather than representing distinct, independently evolving clones. Statistical tests showed no consistent association with suppliers or production/sampling dates (**Supplementary Table S4**). Across both bootstrap thresholds (≥95% and the relaxed ≥85% cutoff), only one clade with 18 isolates was enriched for products produced using potatoes from Farm A (p = 0.010 after FDR), but also included air and surface isolates spanning ~200 days, suggesting persistence rather than supplier origin. No subcluster exhibited significant temporal clustering (p ≥ 0.05 after FDR).

In addition to the lack of supplier or temporal clustering, the spatial context of sampling further supports this interpretation. Environmental isolates sequenced were primarily from the bakery production area, with one isolate originating from the washing/cooking room on a different floor. Combined with the very low SNP divergence, these findings are consistent with a single persistent clonal lineage and support a scenario of long-term environmental persistence as the main driver of contamination, although a more or less continuous introduction from an outside source in which the strain is persistent cannot be entirely excluded. Such persistence suggests that routine cleaning failed to eliminate reservoirs, enabling recurrent contamination of products. Targeted interventions, such as intensified cleaning of high-risk areas (*e.g.*, conveyor belts, air-handling systems) and continuous environmental monitoring, are needed to prevent establishment and dissemination of persistent mould strains.

In dry production environments such as bakeries, the absence of moisture reduces bacterial hazards but does not prevent mould persistence. Spores of xerotolerant fungi, including *Penicillium* spp., can survive on dry surfaces and in flour dust, facilitating airborne dispersal and recolonization despite routine cleaning. Because water use is restricted to avoid creating niches for microbial growth, sanitation relies on dry cleaning methods, which are less effective at completely removing spores (Garcia et al., 2019). These characteristics make persistent contamination particularly challenging to control in bakery settings.

Persistence has been documented for other food-associated microorganisms, including *Listeria monocytogenes*, which can persist for years in processing environments (Fagerlund et al., 2022). Likewise, fungal biofilms on food-contact surfaces are recognized as reservoirs that withstand cleaning and facilitate recontamination (Miranda et al., 2022; Snyder and Worobo, 2018). Our observation that *P. commune* isolates from products and environmental samples cluster together across months suggests analogous persistence mechanisms in moulds. Combined with previous reports on the genomic diversity of *Penicillium* species (Petersen et al., 2023), these findings underscore the value of WGS for strain-level tracking and contamination control in bakery environments.

Mash and SNP-based approaches provided complementary insights into the genetic relatedness of *P. commune* isolates. Mash rapidly placed the 65 bakery isolates into a single clade within the *P. fuscoglaucum*/*P. biforme* lineage and highlighted the distinct position of isolate MF09489. However, Mash distances did not fully reflect fine-scale relationships. SNP analysis, leveraging tens of thousands of high-quality variant sites, revealed extremely low diversity and confirmed long-term persistence of specific genotypes. While Mash is valuable for rapid taxonomic placement, SNP-based phylogenetics is required for high-resolution tracking and inference of contamination dynamics in food production environments. By revealing a single clonal lineage persisting across products and environmental samples, WGS highlights the need to identify contamination hotspots and adapt cleaning protocols accordingly. Measures such as intensified sanitation of conveyor belts, adjustments to air-handling systems, and targeted monitoring of high-risk zones could help prevent re-establishment of persistent strains.

## 4 Conclusions

Whole-genome sequencing provided high-resolution insights into *P. commune* populations in a bakery facility, revealing that isolates from products and the environment belonged to a single clonal lineage persisting for over a year. Traditional barcode markers failed to resolve species boundaries within series *Camembertiorum*, underscoring the need for genome-based identification frameworks.

Statistical analysis showed no consistent clustering by supplier or production date, reinforcing the role of environmental reservoirs as the primary source of contamination. The persistence of nearly identical genotypes across products, air, and surfaces highlights the limitations of routine cleaning and the potential for long-term reservoirs to reseed production lines.

These findings have potential practical implications: in the longer term, WGS-based surveillance could support earlier detection of persistent strains, inform more targeted sanitation strategies, and contribute to reducing mould spoilage and food waste, particularly in facilities with recurrent contamination issues. Furthermore, improving the accuracy of public genome annotations and adopting genome-informed taxonomy will strengthen the reliability of species identification in food safety monitoring.

## Data Availability

The datasets presented in this study can be found in online repositories. The names of the repository/repositories and accession number(s) can be found in the article/**Supplementary Material**.
