# Supplementary material for: Genome-resolved tracking of Penicillium commune in a bakery facility highlights long-term environmental persistence
Source: Front Fungal Biol. 2025 Dec 5;6:1712444. doi: 10.3389/ffunb.2025.1712444 (PMC12714999; doi:10.3389/ffunb.2025.1712444)

Supplementary Material

Genome-resolved tracking of *Penicillium commune* in a bakery facility highlights long-term environmental persistence

Annette Fagerlund*, Charlotte Kummen, Anette Wold Åsli, and Cathrine Kure Finne

Nofima - Norwegian Institute of Food, Fisheries and Aquaculture Research, Ås, Norway

Supplementary Table S1: List of sequenced isolates

NCBI BioSample	MF no	MS no	Species	Sample type	Potato supplier / sampling location	Production / sampling date
SAMN48206909	MF09429	MS0003	Penicillium commune	Product	Farm D	30.09.2019
SAMN48206910	MF09430	MS0006	Penicillium commune	Product	Farm A	03.10.2019
SAMN48206911	MF09431	MS0023	Penicillium commune	Product	Farm D	23.10.2019
SAMN48206912	MF09432	MS0028	Penicillium commune	Product	Farm B	30.10.2019
SAMN48206913	MF09433	MS0031	Penicillium commune	Product	Farm C	25.10.2019
SAMN48206914	MF09434	MS0033	Penicillium commune	Product	Farm C	25.10.2019
SAMN48206915	MF09435	MS0034	Penicillium commune	Product	Farm C	25.10.2019
SAMN48206916	MF09436	MS0036	Penicillium commune	Product	Farm B	08.11.2019
SAMN48206917	MF09437	MS0038	Penicillium commune	Product	Farm B	08.11.2019
SAMN48206918	MF09438	MS0040	Penicillium commune	Product	Farm B	08.11.2019
SAMN48206919	MF09439	MS0042	Penicillium commune	Product	Farm B	11.11.2019
SAMN48206920	MF09440	MS0043	Penicillium commune	Product	Farm B	11.11.2019
SAMN48206921	MF09441	MS0048	Penicillium commune	Product	missing	28.11.2019
SAMN48206922	MF09442	MS0058	Penicillium commune	Air	Potato washing and cooking room	28.11.2019
SAMN48206923	MF09443	MS0076	Penicillium commune	Air	Vacuum cleaner room	28.11.2019
SAMN48206924	MF09444	MS0077	Penicillium rubens	Air	Vacuum cleaner room	28.11.2019
SAMN48206925	MF09445	MS0086	Penicillium commune	Air	Bakery production line 1	04.12.2019
SAMN48206926	MF09446	MS0090	Penicillium commune	Air	Bakery production line 2	04.12.2019
SAMN48206928	MF09448	MS0103	Penicillium commune	Air	Bakery production line 1	06.12.2019
SAMN48206930	MF09450	MS0129	Penicillium commune	Product	Farm A	02.01.2020
SAMN48206931	MF09451	MS0132	Penicillium commune	Product	Farm A	02.01.2020
SAMN48206932	MF09452	MS0135	Penicillium commune	Product	Farm A	03.01.2020
SAMN48206933	MF09453	MS0136	Penicillium commune	Product	Farm A	03.01.2020
SAMN48206934	MF09454	MS0139	Penicillium commune	Product	Farm A	03.01.2020
SAMN48206935	MF09455	MS0141	Penicillium commune	Product	Farm A	03.01.2020
SAMN48206936	MF09456	MS0147	Penicillium commune	Product	Farm A	03.01.2020
SAMN48206937	MF09457	MS0148	Penicillium commune	Product	Farm A	02.01.2020
SAMN48206939	MF09459	MS0158	Penicillium commune	Air	Bakery production line 2	30.01.2020
SAMN48206940	MF09460	MS0160	Penicillium commune	Air	Bakery production line 2	30.01.2020
SAMN48206941	MF09461	MS0163	Penicillium commune	Air	Bakery production line 1	31.01.2020
SAMN48206942	MF09462	MS0164	Penicillium commune	Air	Bakery production line 2	31.01.2020
SAMN48206943	MF09463	MS0165	Penicillium commune	Air	Bakery production line 2	31.01.2020
SAMN48206944	MF09464	MS0169	Penicillium commune	Air	Bakery production line 1	03.02.2020
SAMN48206945	MF09465	MS0171	Penicillium commune	Air	Bakery production line 1	03.02.2020
SAMN48206946	MF09466	MS0172	Penicillium commune	Air	Bakery production line 2	03.02.2020
SAMN48206947	MF09467	MS0175	Penicillium commune	Air	Bakery production line 1	04.02.2020
SAMN48206948	MF09468	MS0177	Penicillium commune	Air	Bakery production line 2	04.02.2020
SAMN48206949	MF09469	MS0182	Penicillium commune	Surface	Conveyor belt	04.02.2020
SAMN48206951	MF09471	MS0185	Penicillium commune	Surface	Conveyor belt	04.02.2020
SAMN48206952	MF09472	MS0186	Penicillium commune	Surface	Conveyor belt	04.02.2020
SAMN48206953	MF09473	MS0187	Penicillium commune	Surface	Conveyor belt	04.02.2020
SAMN48206954	MF09474	MS0188	Penicillium commune	Surface	Robot Picker	04.02.2020
SAMN48206955	MF09475	MS0189	Penicillium commune	Surface	Robot Picker	04.02.2020
SAMN48206956	MF09476	MS0190	Penicillium rubens	Surface	Robot Picker	04.02.2020
SAMN48206957	MF09477	MS0191	Penicillium commune	Surface	Robot Picker	04.02.2020
SAMN48206960	MF09480	MS0196	Penicillium commune	Surface	Conveyor belt	04.02.2020
SAMN48206963	MF09483	MS0199	Penicillium commune	Surface	Conveyor belt	04.02.2020
SAMN48206964	MF09484	MS0200	Penicillium commune	Surface	Conveyor belt	04.02.2020
SAMN48206965	MF09485	MS0202	Penicillium polonicum	Surface	Conveyor belt	04.02.2020
SAMN48206966	MF09486	MS0203	Penicillium commune	Surface	Conveyor belt	04.02.2020
SAMN48206967	MF09487	MS0205	Penicillium commune	Surface	Conveyor belt	04.02.2020
SAMN48206968	MF09488	MS0206	Penicillium commune	Surface	Conveyor belt	04.02.2020

NCBI BioSample	MF no	MS no	Species	Sample type	Potato supplier / sampling location	Production / sampling date
SAMN48206969	MF09489	MS0208	Penicillium commune	Surface	Conveyor belt	04.02.2020
SAMN48206970	MF09490	MS0211	Penicillium commune	Surface	Conveyor belt	04.02.2020
SAMN48206971	MF09491	MS0213	Penicillium commune	Surface	Conveyor belt	04.02.2020
SAMN48206972	MF09492	MS0214	Penicillium commune	Surface	Conveyor belt	04.02.2020
SAMN48206973	MF09493	MS0217	Penicillium commune	Surface	Conveyor belt	04.02.2020
SAMN48206974	MF09494	MS0228	Penicillium commune	Surface	Compressed air	24.10.2019
SAMN48206975	MF09495	MS0262	Penicillium commune	Product	Farm A	23.06.2020
SAMN48206976	MF09496	MS0263	Penicillium commune	Product	Farm A	23.06.2020
SAMN48206977	MF09497	MS0265	Penicillium commune	Product	Farm A	17.06.2020
SAMN48206978	MF09498	MS0266	Penicillium commune	Product	Farm A	17.06.2020
SAMN48206979	MF09499	MS0267	Penicillium commune	Product	Farm A	17.06.2020
SAMN48206980	MF09500	MS0273	Penicillium commune	Product	Farm A	15.06.2020
SAMN48206981	MF09501	MS0275	Penicillium commune	Product	Farm C	19.10.2020
SAMN48206982	MF09502	MS0279	Penicillium commune	Product	Farm B	22.10.2020
SAMN48206983	MF09503	MS0291	Penicillium commune	Product	Farm A	29.12.2020
SAMN48206984	MF09504	MS0296	Penicillium commune	Product	Farm A	05.01.2021

Supplementary Table S2: List of reference genomes

Dataset	Accession no.	Name	Assembly name
Ropars et al. (2020)	SRR12641499	P. biforme ESE00018	
Ropars et al. (2020)	SRR12641514	P. biforme ESE00021	
Ropars et al. (2020)	SRR12641497	P. biforme ESE00023	
Ropars et al. (2020)	SRR12641515	P. biforme ESE00063	
Ropars et al. (2020)	SRR12641496	P. biforme ESE00086	
Ropars et al. (2020)	SRR12641495	P. biforme ESE00087	
Ropars et al. (2020)	SRR12641494	P. biforme ESE00089	
Ropars et al. (2020)	SRR12641492	P. biforme ESE00095	
Ropars et al. (2020)	SRR12641489	P. biforme ESE00125	
Ropars et al. (2020)	SRR12641488	P. biforme ESE00126	
Ropars et al. (2020)	SRR12641487	P. biforme ESE00137	
Ropars et al. (2020)	SRR12641486	P. biforme ESE00139	
Ropars et al. (2020)	SRR12641485	P. biforme ESE00151	
Ropars et al. (2020)	SRR12641483	P. biforme ESE00154	
Ropars et al. (2020)	SRR12641481	P. biforme ESE00156	
Ropars et al. (2020)	SRR12641479	P. biforme ESE00157	
Ropars et al. (2020)	SRR12641478	P. biforme ESE00158	
Ropars et al. (2020)	SRR12641521	P. biforme ESE00202	
Ropars et al. (2020)	SRR12641477	P. biforme ESE00207	
Ropars et al. (2020)	SRR12641476	P. biforme ESE00211	
Ropars et al. (2020)	SRR12641535	P. biforme ESE00213=CBS 297.48T	
Ropars et al. (2020)	SRR12641526	P. biforme ESE00215 - P. commune CBS 216.30NT	
Ropars et al. (2020)	SRR12641525	P. biforme ESE00217	
Ropars et al. (2020)	SRR12641528	P. biforme ESE00222	
Ropars et al. (2020)	SRR12641509	P. biforme ESE00223	
Ropars et al. (2020)	SRR12641507	P. biforme ESE00228	
Ropars et al. (2020)	SRR12641508	P. biforme ESE00239	
Ropars et al. (2020)	SRR12641500	P. biforme ESE00241	
Ropars et al. (2020)	SRR12641517	P. camemberti var. camemberti ESE00061	
Ropars et al. (2020)	SRR12641532	P. camemberti var. camemberti ESE00203	
Ropars et al. (2020)	SRR12641480	P. camemberti var. camemberti ESE00204=LCP00584T	
Ropars et al. (2020)	SRR12641534	P. camemberti var. camemberti ESE00205	
Ropars et al. (2020)	SRR12641527	P. camemberti var. camemberti ESE00219	
Ropars et al. (2020)	SRR12641511	P. camemberti var. camemberti ESE00227	
Ropars et al. (2020)	SRR12641505	P. camemberti var. camemberti ESE00240	
Ropars et al. (2020)	SRR12641512	P. camemberti var. camemberti ESE00242	
Ropars et al. (2020)	SRR12641506	P. camemberti var. camemberti ESE00243	
Ropars et al. (2020)	SRR12641501	P. camemberti var. camemberti ESE00244	
Ropars et al. (2020)	SRR12641504	P. camemberti var. camemberti ESE00246	
Ropars et al. (2020)	SRR12641503	P. camemberti var. camemberti ESE00247	
Ropars et al. (2020)	SRR12641498	P. camemberti var. caseifulvum ESE00019	
Ropars et al. (2020)	SRR12641490	P. camemberti var. caseifulvum ESE00120	
Ropars et al. (2020)	SRR12641519	P. camemberti var. caseifulvum ESE00214	
Ropars et al. (2020)	SRR12641474	P. camemberti var. caseifulvum ESE00224	
Ropars et al. (2020)	SRR12641513	P. camemberti var. caseifulvum ESE00226=LCP05630T	
Ropars et al. (2020)	SRR12641510	P. camemberti var. caseifulvum ESE00245	
Ropars et al. (2020)	SRR12641524	P. fuscoglaucum ESE00059 - P. commune CBS 111835	
Ropars et al. (2020)	SRR12641516	P. fuscoglaucum ESE00062	
Ropars et al. (2020)	SRR12641493	P. fuscoglaucum ESE00090	
Ropars et al. (2020)	SRR12641482	P. fuscoglaucum ESE00155	
Ropars et al. (2020)	SRR12641518	P. fuscoglaucum ESE00199	
Ropars et al. (2020)	SRR12641522	P. fuscoglaucum ESE00200	
Ropars et al. (2020)	SRR12641491	P. fuscoglaucum ESE00208	
Ropars et al. (2020)	SRR12641523	P. fuscoglaucum ESE00209	
Ropars et al. (2020)	SRR12641530	P. fuscoglaucum ESE00210	
Ropars et al. (2020)	SRR12641520	P. fuscoglaucum ESE00212 - P. commune CBS 112079	
Ropars et al. (2020)	SRR12641533	P. fuscoglaucum ESE00216	
Ropars et al. (2020)	SRR12641475	P. fuscoglaucum ESE00218	
Ropars et al. (2020)	SRR12641529	P. fuscoglaucum ESE00220	
Ropars et al. (2020)	SRR12641473	P. fuscoglaucum ESE00221	
Ropars et al. (2020)	SRR12641484	P. palitans ESE00153	
GenBank/RefSeq	GCA_028826965.1	P. alfredii IBT 34128	GCA_028826965.1_ASM2882696v1
GenBank/RefSeq	GCA_028827245.1	P. angulare IBT 27051	GCA_028827245.1_ASM2882724v1
GenBank/RefSeq	GCA_028974205.1	P. antarcticum IBT 31339	GCA_028974205.1_ASM2897420v1
GenBank/RefSeq	GCA_028826775.1	P. argentinense IBT 30761	GCA_028826775.1_ASM2882677v1
GenBank/RefSeq	GCA_001773325.1	P. arizonense CBS 141311	GCA_001773325.1_ASM177332v1
GenBank/RefSeq	GCA_028827265.1	P. atrosanguineum IBT 20685	GCA_028827265.1_ASM2882726v1

Dataset	Accession no.	Name	Assembly name
GenBank/RefSeq	GCA_019977855.1	P. aurantiogriseum IBT 35659	GCA_019977855.1_ASM1997785v1
GenBank/RefSeq	GCA_026122735.1	P. bialowiezense A30	GCA_026122735.1_ASM2612273v1
GenBank/RefSeq	GCA_000577785.1	P. biforme FM169	GCA_000577785.1_PBIFFM169_20131217
GenBank/RefSeq	GCA_028826915.1	P. bovifimosum IBT 22155	GCA_028826915.1_ASM2882691v1
GenBank/RefSeq	GCA_001048715.1	P. brasilianum	GCA_001048715.1_Pbras_Allpaths-LG
GenBank/RefSeq	GCA_040333145.1	P. brefeldianum F032	GCA_040333145.1_ASM4033314v1
GenBank/RefSeq	GCA_028827555.1	P. brevicompactum IBT 35665	GCA_028827555.1_ASM2882755v1
GenBank/RefSeq	GCA_014839975.1	P. camemberti FM013=LCP06093	GCA_014839975.1_PcamFM013r2_polished
GenBank/RefSeq	GCA_028826845.1	P. canariense IBT 26290	GCA_028826845.1_ASM2882684v1
GenBank/RefSeq	GCA_028828765.1	P. canescens IBT 15451	GCA_028828765.1_ASM2882876v1
GenBank/RefSeq	GCA_028828875.1	P. capsulatum IBT 29712	GCA_028828875.1_ASM2882887v1
GenBank/RefSeq	GCA_000577495.1	P. carneum LCP05634	GCA_000577495.1_PCARLCP05634_20131217
GenBank/RefSeq	GCA_022813165.1	P. caseifulvum FKI-L3-CM-P1	GCA_022813165.1_ASM2281316v1
GenBank/RefSeq	GCA_028827025.1	P. cataractarum IBT 29864	GCA_028827025.1_ASM2882702v1
GenBank/RefSeq	GCA_028974085.1	P. chermesinum IBT 19713	GCA_028974085.1_ASM2897408v1
GenBank/RefSeq	GCA_028827035.1	P. chrysogenum IBT 35668	GCA_028827035.1_ASM2882703v1
GenBank/RefSeq	GCA_028974065.1	P. cinerascens IBT 15544	GCA_028974065.1_ASM2897406v1
GenBank/RefSeq	GCA_028827155.1	P. citrinum IBT 23319	GCA_028827155.1_ASM2882715v1
GenBank/RefSeq	GCA_035985395.1	P. coffeae ANU01	GCA_035985395.1_ASM3598539v1
GenBank/RefSeq	GCA_028827145.1	P. concentricum IBT 3081	GCA_028827145.1_ASM2882714v1
GenBank/RefSeq	GCA_028826855.1	P. coprophilum IBT 35676	GCA_028826855.1_ASM2882685v1
GenBank/RefSeq	GCA_018410145.1	P. corylophilum F5_1S_1A_F	GCA_018410145.1_ASM1841014v1
GenBank/RefSeq	GCA_028827165.1	P. cosmopolitanum IBT 29677	GCA_028827165.1_ASM2882716v1
GenBank/RefSeq	GCA_028827405.1	P. crustosum IBT 35664	GCA_028827405.1_ASM2882740v1
GenBank/RefSeq	GCA_027569675.1	P. crystallinum NRRL 5082	GCA_027569675.1_ASM2756967v1
GenBank/RefSeq	GCA_028827525.1	P. daleae IBT 16125	GCA_028827525.1_ASM2882752v1
GenBank/RefSeq	GCA_027569385.1	P. decumbens NRRL 741	GCA_027569385.1_ASM2756938v1
GenBank/RefSeq	GCA_028827375.1	P. desertorum IBT 17660	GCA_028827375.1_ASM2882737v1
GenBank/RefSeq	GCA_028827545.1	P. diatomitis IBT 30728	GCA_028827545.1_ASM2882754v1
GenBank/RefSeq	GCA_016767815.1	P. digitatum PdW03	GCA_016767815.1_ASM1676781v1
GenBank/RefSeq	GCA_015585785.1	P. dipodomyicola IIF7SW-F2	GCA_015585785.1_ASM1558578v1
GenBank/RefSeq	GCA_902713485.1	P. discolor 3B6	GCA_902713485.1_Pdiscolor_3B6_v2
GenBank/RefSeq	GCA_911456345.1	P. egyptiacum LCP06446	GCA_911456345.1_Pegy_LCP06446
GenBank/RefSeq	GCA_000769745.1	P. expansum MD-8	GCA_000769745.1_ASM76974v1
GenBank/RefSeq	GCA_028828255.1	P. fimorum IBT 29495	GCA_028828255.1_ASM2882825v1
GenBank/RefSeq	GCA_002072365.1	P. flavigenum IBT 14082	GCA_002072365.1_ASM207236v1
GenBank/RefSeq	GCA_028827385.1	P. freii IBT 34325	GCA_028827385.1_ASM2882738v1
GenBank/RefSeq	GCA_028828275.1	P. frequentans IBT 35677	GCA_028828275.1_ASM2882827v1
GenBank/RefSeq	GCA_037044005.1	P. fructuariae-cellae LH_A412	GCA_037044005.1_ASM3704400v1
GenBank/RefSeq	GCA_040250155.1	P. fuscoglaucum Pf_T2	GCA_040250155.1_Pf_T2
GenBank/RefSeq	GCA_037040985.1	P. glabrum DX136-07W	GCA_037040985.1_ASM3704098v1
GenBank/RefSeq	GCA_902713505.1	P. glandicola 3C	GCA_902713505.1_Pglandicola_3C_v2
GenBank/RefSeq	GCA_025586815.1	P. glycyrrhizacola CGMCC 3.15273	GCA_025586815.1_ASM2558681v1
GenBank/RefSeq	GCA_037043885.1	P. goetzii LH_A469	GCA_037043885.1_ASM3704388v1
GenBank/RefSeq	GCA_001561935.1	P. griseofulvum PG3	GCA_001561935.1_ASM156193v1
GenBank/RefSeq	GCA_015586035.1	P. griseoroseum IF3SW-F1	GCA_015586035.1_ASM1558603v1
GenBank/RefSeq	GCA_039634405.1	P. herquei HGN12.1C (12C)	GCA_039634405.1_ASM3963440v1
GenBank/RefSeq	GCA_028827645.1	P. hetheringtonii IBT 29057	GCA_028827645.1_ASM2882764v1
GenBank/RefSeq	GCA_028827665.1	P. hispanicum IBT 35686	GCA_028827665.1_ASM2882766v1
GenBank/RefSeq	GCA_028827395.1	P. hordei IBT 12815	GCA_028827395.1_ASM2882739v1
GenBank/RefSeq	GCA_002116305.1	P. italicum GL-Gan1	GCA_002116305.1_ASM211630v1
GenBank/RefSeq	GCA_027569415.1	P. janthinellum NRRL 35451	GCA_027569415.1_ASM2756941v1
GenBank/RefSeq	GCA_050613445.1	P. koreense GX2	GCA_050613445.1_ASM5061344v1
GenBank/RefSeq	GCA_028827675.1	P. lagena IBT 129212	GCA_028827675.1_ASM2882767v1
GenBank/RefSeq	GCA_028828245.1	P. lividum IBT 13676	GCA_028828245.1_ASM2882824v1
GenBank/RefSeq	GCA_028827895.1	P. longicatenatum IBT 33135	GCA_028827895.1_ASM2882789v1
GenBank/RefSeq	GCA_028827695.1	P. maclennaniae IBT 15551	GCA_028827695.1_ASM2882769v1
GenBank/RefSeq	GCA_028827735.1	P. macrosclerotiorum IBT 26536	GCA_028827735.1_ASM2882773v1
GenBank/RefSeq	GCA_028828285.1	P. majusculum IBT 35410	GCA_028828285.1_ASM2882828v1
GenBank/RefSeq	GCA_028827825.1	P. malachiteum IBT 17515	GCA_028827825.1_ASM2882782v1
GenBank/RefSeq	GCA_028828005.1	P. manginii IBT 31320	GCA_028828005.1_ASM2882800v1
GenBank/RefSeq	GCA_028829835.1	P. mononematosum IBT 11891	GCA_028829835.1_ASM2882983v1
GenBank/RefSeq	GCA_911456355.1	P. nalgiovense ESE00252	GCA_911456355.1_Pnal_ESE00252
GenBank/RefSeq	GCA_000733025.2	P. nordicum UASWS BFE487	GCA_000733025.2_PnBFE487-1.0
GenBank/RefSeq	GCA_028828085.1	P. nucicola IBT 29836	GCA_028828085.1_ASM2882808v1
GenBank/RefSeq	GCA_002382855.1	P. occitanis (nom. inval.) CL100	GCA_002382855.1_ASM238285v1
GenBank/RefSeq	GCA_022985105.1	P. ochrochloron RLS11	GCA_022985105.1_UFV_RLS11_1.0
GenBank/RefSeq	GCA_028828045.1	P. odoratum IBT 22623	GCA_028828045.1_ASM2882804v1
GenBank/RefSeq	GCA_911174995.1	P. olsonii LCP05357	GCA_911174995.1_Pols_LCP05357
GenBank/RefSeq	GCA_001723175.3	P. oxalicum HP7-1	GCA_001723175.3_ASM172317v3

Dataset	Accession no.	Name	Assembly name
GenBank/RefSeq	GCA_019190355.1	P. palitans F6_7S_1C_F	GCA_019190355.1_ASM1919035v1
GenBank/RefSeq	GCA_902713525.1	P. pancosmium FP10	GCA_902713525.1_Ppancosmium_FP1_v2
GenBank/RefSeq	GCA_023627375.1	P. paneum M1707	GCA_023627375.1_ASM2362737v1
GenBank/RefSeq	GCA_028828445.1	P. paradoxum IBT 22861	GCA_028828445.1_ASM2882844v1
GenBank/RefSeq	GCA_030035635.1	P. parvum 4-14b	GCA_030035635.1_ASM3003563v1
GenBank/RefSeq	GCA_000347475.1	P. paxilli ATCC 26601	GCA_000347475.1_Ppaxilli_v.1.0
GenBank/RefSeq	GCA_034423695.1	P. polonicum KACC_93368	GCA_034423695.1_ASM3442369v1
GenBank/RefSeq	GCA_964197705.1	P. psychrofluorescens KMR99	GCA_964197705.1_Pfluo_KMR99
GenBank/RefSeq	GCA_028828465.1	P. psychrosexuale IBT 29551	GCA_028828465.1_ASM2882846v1
GenBank/RefSeq	GCA_028828015.1	P. pulvis IBT 33274	GCA_028828015.1_ASM2882801v1
GenBank/RefSeq	GCA_040333315.2	P. raperi F027	GCA_040333315.2_ASM4033331v2
GenBank/RefSeq	GCA_028828495.1	P. riverlandense IBT 135883	GCA_028828495.1_ASM2882849v1
GenBank/RefSeq	GCA_028829455.1	P. robsamsonii IBT 29466	GCA_028829455.1_ASM2882945v1
GenBank/RefSeq	GCA_011392555.1	P. rolfsii F1880	GCA_011392555.1_CTC_F1880_1.0
GenBank/RefSeq	GCA_015533775.1	P. roqueforti LCP96 04111	GCA_015533775.1_ASM1553377v1
GenBank/RefSeq	GCA_028828025.1	P. rubens IBT 27055	GCA_028828025.1_ASM2882802v1
GenBank/RefSeq	GCA_911197225.1	P. salamii DTO309-F9	GCA_911197225.1_Psal_DTO309-F9
GenBank/RefSeq	GCA_028829775.1	P. samsonianum IBT 33392	GCA_028829775.1_ASM2882977v1
GenBank/RefSeq	GCA_928213465.1	P. sclerotigenum IBT15061	GCA_928213465.1_Penicillium_sclerotigenum_IBT15061
GenBank/RefSeq	GCA_911649655.1	P. sclerotiorum wengan_M_CN111	CN111
GenBank/RefSeq	GCA_027569585.1	P. silybi G85	GCA_027569585.1_ASM2756958v1
GenBank/RefSeq	GCA_024706585.1	P. simplicissimum A4	GCA_024706585.1_ASM2470658v1
GenBank/RefSeq	GCA_028829755.1	P. solitum IBT 25940	GCA_028829755.1_ASM2882975v1
GenBank/RefSeq	GCA_028829465.1	P. soppii IBT 18220	GCA_028829465.1_ASM2882946v1
GenBank/RefSeq	GCA_018340795.1	P. steckii P2648	GCA_018340795.1_PS_2648
GenBank/RefSeq	GCA_028828155.1	P. subrubescens IBT 31985	GCA_028828155.1_ASM2882815v1
GenBank/RefSeq	GCA_020086695.1	P. sumatraense AQ67100	GCA_020086695.1_ASM2008669v1
GenBank/RefSeq	GCA_028829675.1	P. tannophilum IBT 21756	GCA_028829675.1_ASM2882967v1
GenBank/RefSeq	GCA_028828555.1	P. taxi IBT 34144	GCA_028828555.1_ASM2882855v1
GenBank/RefSeq	GCA_030142185.1	P. thymicola DAOM 180753	GCA_030142185.1_ASM3014218v1
GenBank/RefSeq	GCA_030762825.1	P. turbatum BLH34	GCA_030762825.1_ASM3076282v1
GenBank/RefSeq	GCA_014839625.1	P. ucsense S1M29	GCA_014839625.1_UCS_PECM_1.0
GenBank/RefSeq	GCA_028828195.1	P. verhagenii IBT 33310	GCA_028828195.1_ASM2882819v1
GenBank/RefSeq	GCA_028828655.1	P. verrucosum IBT 35672	GCA_028828655.1_ASM2882865v1
GenBank/RefSeq	GCA_028828185.1	P. viridicatum IBT 34249	GCA_028828185.1_ASM2882818v1
GenBank/RefSeq	GCA_028829585.1	P. vulpinum IBT 29486	GCA_028829585.1_ASM2882958v1
GenBank/RefSeq	GCA_028829765.1	P. waksmanii IBT 27052	GCA_028829765.1_ASM2882976v1
GenBank	GCA_000230395.2	Aspergillus niger ATCC 1015	GCA_000230395.2_ASPNI_v3.0
GenBank	GCA_028826755.1	P. angulare IBT 30069	GCA_028826755.1_ASM2882675v1
GenBank	GCA_002072345.1	P. antarcticum IBT 31811	GCA_002072345.1_ASM207234v1
GenBank	GCA_028827005.1	P. atrosanguineum IBT 21472	GCA_028827005.1_ASM2882700v1
GenBank	GCA_028826785.1	P. atrosanguineum IBT 31632	GCA_028826785.1_ASM2882678v1
GenBank	GCA_028826765.1	P. atrosanguineum IBT 34669	GCA_028826765.1_ASM2882676v1
GenBank	GCA_902713515.1	P. bialowiezense CAS30	GCA_902713515.1_Pbialowiezense_CAS30_v2
GenBank	GCA_002016555.1	P. brasilianum LaBioMMi 136	GCA_002016555.1_ASM201655v1
GenBank	GCA_004916855.1	P. brasilianum ZJ-7	GCA_004916855.1_ASM491685v1
GenBank	GCA_040333375.2	P. brefeldianum F015	GCA_040333375.2_ASM4033337v2
GenBank	GCA_040144005.1	P. brevicompactum CMG72	GCA_040144005.1_ASM4014400v1
GenBank	GCA_022813345.1	P. brevicompactum FJII-L9-SW-P1	GCA_022813345.1_ASM2281334v1
GenBank	GCA_019843585.1	P. brevicompactum HOCR17	GCA_019843585.1_ASM1984358v1
GenBank	GCA_028827255.1	P. brevicompactum IBT 35673	GCA_028827255.1_ASM2882725v1
GenBank	GCA_028827515.1	P. brevicompactum IBT 35675	GCA_028827515.1_ASM2882751v1
GenBank	GCA_015586265.1	P. camemberti F3_3F1_F	GCA_015586265.1_ASM1558626v1
GenBank	GCA_000513335.1	P. camemberti FM 013	GCA_000513335.1_PCAMFM013_20131106
GenBank	GCA_015586095.1	P. camemberti IF2SW-F1	GCA_015586095.1_ASM1558609v1
GenBank	GCA_015585745.1	P. camemberti IIF8SW-F2	GCA_015585745.1_ASM1558574v1
GenBank	GCA_015585755.1	P. camemberti IIF8SW-F3	GCA_015585755.1_ASM1558575v1
GenBank	GCA_028829005.1	P. canescens IBT 13549	GCA_028829005.1_ASM2882900v1
GenBank	GCA_028828745.1	P. canescens IBT 15449	GCA_028828745.1_ASM2882874v1
GenBank	GCA_028828735.1	P. canescens IBT 15450	GCA_028828735.1_ASM2882873v1
GenBank	GCA_028829745.1	P. canescens IBT 15452	GCA_028829745.1_ASM2882974v1
GenBank	GCA_028828795.1	P. canescens IBT 18980	GCA_028828795.1_ASM2882879v1
GenBank	GCA_028828825.1	P. canescens IBT 19259	GCA_028828825.1_ASM2882882v1
GenBank	GCA_000943765.1	P. capsulatum ATCC 48735	GCA_000943765.1_ASM94376v1
GenBank	GCA_028826875.1	P. capsulatum IBT 21917	GCA_028826875.1_ASM2882687v1
GenBank	GCA_000943775.1	P. capsulatum LiaoWQ-2011	GCA_000943775.1_ASM94377v1
GenBank	GCA_022813545.1	P. caseifulvum FJII-L4-SW-DR1	GCA_022813545.1_ASM2281354v1
GenBank	GCA_019828795.1	P. cf. chrysogenum S/N-302-OC-P1	GCA_019828795.1_ASM1982879v1
GenBank	GCA_019828875.1	P. cf. chrysogenum S/N-302-OC-R1	GCA_019828875.1_ASM1982887v1
GenBank	GCA_019827435.1	P. cf. chrysogenum S/N-302-OC-R3	GCA_019827435.1_ASM1982743v1

Dataset	Accession no.	Name	Assembly name
GenBank	GCA_020284065.1	P. cf. chrysogenum S/N-308-OC-R5	GCA_020284065.1_ASM2028406v1
GenBank	GCA_028826995.1	P. cf. griseofulvum IBT 16848	GCA_028826995.1_ASM2882699v1
GenBank	GCA_028974015.1	P. cf. griseofulvum IBT 16849	GCA_028974015.1_ASM2897401v1
GenBank	GCA_028827235.1	P. cf. griseofulvum IBT 17755	GCA_028827235.1_ASM2882723v1
GenBank	GCA_028974045.1	P. cf. viridicatum IBT 20477	GCA_028974045.1_ASM2897404v1
GenBank	GCA_028828985.1	P. chermesinum IBT 22717	GCA_028828985.1_ASM2882898v1
GenBank	GCA_023626975.1	P. chrysogenum 2NP912A	GCA_023626975.1_ASM2362697v1
GenBank	GCA_044231865.1	P. chrysogenum 404	GCA_044231865.1_ASM4423186v1
GenBank	GCA_044231845.1	P. chrysogenum 413	GCA_044231845.1_ASM4423184v1
GenBank	GCA_023626635.1	P. chrysogenum B20-02	GCA_023626635.1_ASM2362663v1
GenBank	GCA_023626855.1	P. chrysogenum B3902	GCA_023626855.1_ASM2362685v1
GenBank	GCA_028891605.1	P. chrysogenum BIONCL16	GCA_028891605.1_ASM2889160v1
GenBank	GCA_048127645.1	P. chrysogenum BIONCL16	GCA_048127645.1_ASM4812764v1
GenBank	GCA_025590035.1	P. chrysogenum CGMCC 3.15265	GCA_025590035.1_ASM2559003v1
GenBank	GCA_015586315.1	P. chrysogenum F3_2F3_F	GCA_015586315.1_ASM1558631v1
GenBank	GCA_015586305.1	P. chrysogenum F3_2F4_F	GCA_015586305.1_ASM1558630v1
GenBank	GCA_015586295.1	P. chrysogenum F3_2F5_F	GCA_015586295.1_ASM1558629v1
GenBank	GCA_023627295.1	P. chrysogenum F30-04	GCA_023627295.1_ASM2362729v1
GenBank	GCA_002080375.1	P. chrysogenum HKF42	GCA_002080375.1_ASM208037v1
GenBank	GCA_000801355.1	P. chrysogenum IB 08/921	GCA_000801355.1_ASM80135v1
GenBank	GCA_028828955.1	P. chrysogenum IBT 17219	GCA_028828955.1_ASM2882895v1
GenBank	GCA_028826945.1	P. chrysogenum IBT 19737	GCA_028826945.1_ASM2882694v1
GenBank	GCA_028827015.1	P. chrysogenum IBT 3361	GCA_028827015.1_ASM2882701v1
GenBank	GCA_015586425.1	P. chrysogenum IF1SG-B2	GCA_015586425.1_ASM1558642v1
GenBank	GCA_015586135.1	P. chrysogenum IF1SW-F3	GCA_015586135.1_ASM1558613v1
GenBank	GCA_015586415.1	P. chrysogenum IF2SG-B2	GCA_015586415.1_ASM1558641v1
GenBank	GCA_015586075.1	P. chrysogenum IF2SW-F4	GCA_015586075.1_ASM1558607v1
GenBank	GCA_015586055.1	P. chrysogenum IF2SW-F5	GCA_015586055.1_ASM1558605v1
GenBank	GCA_015586015.1	P. chrysogenum IF3SW-F3	GCA_015586015.1_ASM1558601v1
GenBank	GCA_015586395.1	P. chrysogenum IF4SG-B1	GCA_015586395.1_ASM1558639v1
GenBank	GCA_015585965.1	P. chrysogenum IF4SW-F1	GCA_015585965.1_ASM1558596v1
GenBank	GCA_015585955.1	P. chrysogenum IF7SW-F1	GCA_015585955.1_ASM1558595v1
GenBank	GCA_015586365.1	P. chrysogenum IIF2*SW-F2	GCA_015586365.1_ASM1558636v1
GenBank	GCA_015585875.1	P. chrysogenum IIF3SW-F2	GCA_015585875.1_ASM1558587v1
GenBank	GCA_015585855.1	P. chrysogenum IIF4SW-F3	GCA_015585855.1_ASM1558585v1
GenBank	GCA_015585735.1	P. chrysogenum IIF8SW-F4	GCA_015585735.1_ASM1558573v1
GenBank	GCA_000816005.1	P. chrysogenum KF-25	GCA_000816005.1_KF-25_v1
GenBank	GCA_023626815.1	P. chrysogenum M20-01	GCA_023626815.1_ASM2362681v1
GenBank	GCA_023627355.1	P. chrysogenum M20-02	GCA_023627355.1_ASM2362735v1
GenBank	GCA_023624315.1	P. chrysogenum M2201	GCA_023624315.1_ASM2362431v1
GenBank	GCA_023627315.1	P. chrysogenum M2202	GCA_023627315.1_ASM2362731v1
GenBank	GCA_025768175.1	P. chrysogenum M2203	GCA_025768175.1_ASM2576817v1
GenBank	GCA_023627035.1	P. chrysogenum M30-01	GCA_023627035.1_ASM2362703v1
GenBank	GCA_000523475.1	P. chrysogenum NCPC10086	GCA_000523475.1_Penc1.0
GenBank	GCA_027569345.1	P. chrysogenum NRRL 792	GCA_027569345.1_ASM2756934v1
GenBank	GCA_023624235.1	P. chrysogenum P20-02	GCA_023624235.1_ASM2362423v1
GenBank	GCA_023624435.1	P. chrysogenum P20-04	GCA_023624435.1_ASM2362443v1
GenBank	GCA_000710275.1	P. chrysogenum P2niaD18	GCA_000710275.1_ASM71027v1
GenBank	GCA_023624455.1	P. chrysogenum P30-11	GCA_023624455.1_ASM2362445v1
GenBank	GCA_023626755.1	P. chrysogenum PA3101	GCA_023626755.1_ASM2362675v1
GenBank	GCA_023626715.1	P. chrysogenum PB20-03	GCA_023626715.1_ASM2362671v1
GenBank	GCA_025768475.1	P. chrysogenum PB20-04	GCA_025768475.1_ASM2576847v1
GenBank	GCA_023627195.1	P. chrysogenum PB2409	GCA_023627195.1_ASM2362719v1
GenBank	GCA_023624375.1	P. chrysogenum PB3102	GCA_023624375.1_ASM2362437v1
GenBank	GCA_023626675.1	P. chrysogenum PB3103	GCA_023626675.1_ASM2362667v1
GenBank	GCA_023626655.1	P. chrysogenum PB3904	GCA_023626655.1_ASM2362665v1
GenBank	GCA_023627015.1	P. chrysogenum PDH20-03	GCA_023627015.1_ASM2362701v1
GenBank	GCA_023626995.1	P. chrysogenum PDH20-04	GCA_023626995.1_ASM2362699v1
GenBank	GCA_023626795.1	P. chrysogenum PF2508S	GCA_023626795.1_ASM2362679v1
GenBank	GCA_023624355.1	P. chrysogenum PF3401	GCA_023624355.1_ASM2362435v1
GenBank	GCA_023627255.1	P. chrysogenum PG3507	GCA_023627255.1_ASM2362725v1
GenBank	GCA_023626735.1	P. chrysogenum PG4202	GCA_023626735.1_ASM2362673v1
GenBank	GCA_023627215.1	P. chrysogenum PH2403	GCA_023627215.1_ASM2362721v1
GenBank	GCA_023626695.1	P. chrysogenum PH4103	GCA_023626695.1_ASM2362669v1
GenBank	GCA_023627055.1	P. chrysogenum PK3604	GCA_023627055.1_ASM2362705v1
GenBank	GCA_023624415.1	P. chrysogenum PL40-01	GCA_023624415.1_ASM2362441v1
GenBank	GCA_023624255.1	P. chrysogenum PM3404	GCA_023624255.1_ASM2362425v1
GenBank	GCA_023626595.1	P. chrysogenum PYS3203	GCA_023626595.1_ASM2362659v1
GenBank	GCA_023624295.1	P. chrysogenum R1210	GCA_023624295.1_ASM2362429v1
GenBank	GCA_023626775.1	P. chrysogenum R1211B	GCA_023626775.1_ASM2362677v1

Dataset	Accession no.	Name	Assembly name
GenBank	GCA_023627175.1	P. chrysogenum R13B	GCA_023627175.1_ASM2362717v1
GenBank	GCA_023626835.1	P. chrysogenum R20-04	GCA_023626835.1_ASM2362683v1
GenBank	GCA_023627135.1	P. chrysogenum R20-05	GCA_023627135.1_ASM2362713v1
GenBank	GCA_023624395.1	P. chrysogenum R20-08	GCA_023624395.1_ASM2362439v1
GenBank	GCA_023624165.1	P. chrysogenum R2501	GCA_023624165.1_ASM2362416v1
GenBank	GCA_023624185.1	P. chrysogenum R3104	GCA_023624185.1_ASM2362418v1
GenBank	GCA_023626915.1	P. chrysogenum R3301	GCA_023626915.1_ASM2362691v1
GenBank	GCA_023627275.1	P. chrysogenum R3406	GCA_023627275.1_ASM2362727v1
GenBank	GCA_023624275.1	P. chrysogenum R4101	GCA_023624275.1_ASM2362427v1
GenBank	GCA_023624335.1	P. chrysogenum R4403	GCA_023624335.1_ASM2362433v1
GenBank	GCA_023626935.1	P. chrysogenum S1301	GCA_023626935.1_ASM2362693v1
GenBank	GCA_023626955.1	P. chrysogenum S1302	GCA_023626955.1_ASM2362695v1
GenBank	GCA_023627335.1	P. chrysogenum S20-02	GCA_023627335.1_ASM2362733v1
GenBank	GCA_023626895.1	P. chrysogenum S2404	GCA_023626895.1_ASM2362689v1
GenBank	GCA_023627155.1	P. chrysogenum S2406	GCA_023627155.1_ASM2362715v1
GenBank	GCA_023626875.1	P. chrysogenum S3404	GCA_023626875.1_ASM2362687v1
GenBank	GCA_023627095.1	P. chrysogenum S3406	GCA_023627095.1_ASM2362709v1
GenBank	GCA_023627115.1	P. chrysogenum S40-01	GCA_023627115.1_ASM2362711v1
GenBank	GCA_023627235.1	P. chrysogenum X20-07	GCA_023627235.1_ASM2362723v1
GenBank	GCA_023627075.1	P. chrysogenum Y1301	GCA_023627075.1_ASM2362707v1
GenBank	GCA_025768145.1	P. chrysogenum Y3303	GCA_025768145.1_ASM2576814v1
GenBank	GCA_023624135.1	P. chrysogenum Y40-02	GCA_023624135.1_ASM2362413v1
GenBank	GCA_025768765.1	P. citrinum B8014	GCA_025768765.1_ASM2576876v1
GenBank	GCA_036320845.1	P. citrinum B9	GCA_036320845.1_ASM3632084v1
GenBank	GCA_001399475.1	P. citrinum DSM 1997	GCA_001399475.1_PCAss
GenBank	GCA_019191195.1	P. citrinum F4_1A_F1_F	GCA_019191195.1_ASM1919119v1
GenBank	GCA_001950535.1	P. citrinum JCM 22607	GCA_001950535.1_JCM_22607_assembly_v001
GenBank	GCA_037044215.1	P. citrinum LH_A494	GCA_037044215.1_ASM3704421v1
GenBank	GCA_025768525.1	P. citrinum M7005D	GCA_025768525.1_ASM2576852v1
GenBank	GCA_020284165.1	P. citrinum NRRL 1841	GCA_020284165.1_ASM2028416v1
GenBank	GCA_027569755.1	P. citrinum NRRL 756	GCA_027569755.1_ASM2756975v1
GenBank	GCA_025531905.1	P. citrinum P8002	GCA_025531905.1_ASM2553190v1
GenBank	GCA_023624655.1	P. citrinum P8015	GCA_023624655.1_ASM2362465v1
GenBank	GCA_025768325.1	P. citrinum PA2506	GCA_025768325.1_ASM2576832v1
GenBank	GCA_025768585.1	P. citrinum PA2509	GCA_025768585.1_ASM2576858v1
GenBank	GCA_023624675.1	P. citrinum PA2510	GCA_023624675.1_ASM2362467v1
GenBank	GCA_025768705.1	P. citrinum PB2504	GCA_025768705.1_ASM2576870v1
GenBank	GCA_025768355.1	P. citrinum PB2505	GCA_025768355.1_ASM2576835v1
GenBank	GCA_025768795.1	P. citrinum PDH2504	GCA_025768795.1_ASM2576879v1
GenBank	GCA_025768615.1	P. citrinum PF2509S	GCA_025768615.1_ASM2576861v1
GenBank	GCA_025768735.1	P. citrinum PG2501	GCA_025768735.1_ASM2576873v1
GenBank	GCA_023624595.1	P. citrinum PH4033	GCA_023624595.1_ASM2362459v1
GenBank	GCA_025768665.1	P. citrinum PT2503	GCA_025768665.1_ASM2576866v1
GenBank	GCA_025768405.1	P. citrinum PT2504	GCA_025768405.1_ASM2576840v1
GenBank	GCA_025768445.1	P. citrinum PT2506	GCA_025768445.1_ASM2576844v1
GenBank	GCA_025768385.1	P. citrinum PYS008B	GCA_025768385.1_ASM2576838v1
GenBank	GCA_025782865.1	P. citrinum R2503	GCA_025782865.1_ASM2578286v1
GenBank	GCA_025768645.1	P. citrinum S2504	GCA_025768645.1_ASM2576864v1
GenBank	GCA_025768555.1	P. citrinum X2502D	GCA_025768555.1_ASM2576855v1
GenBank	GCA_002072405.1	P. coprophilum IBT 31321	GCA_002072405.1_ASM207240v1
GenBank	GCA_027569365.1	P. coprophilum NRRL 13627	GCA_027569365.1_ASM2756936v1
GenBank	GCA_018410175.1	P. corylophilum F5_1S_1B_F	GCA_018410175.1_ASM1841017v1
GenBank	GCA_902712905.1	P. crustosum CAL64	GCA_902712905.1_Pcrustosum_CAL64_v2
GenBank	GCA_014621375.1	P. crustosum G10	GCA_014621375.1_ASM1462137v1
GenBank	GCA_025583935.1	P. crustosum IFST Pcru1	GCA_025583935.1_ASM2558393v1
GenBank	GCA_043643955.1	P. crustosum PRB-2	GCA_043643955.1_ASM4364395v1
GenBank	GCA_022813305.1	P. decumbens FJII-L3-CM-PAB4	GCA_022813305.1_ASM2281330v1
GenBank	GCA_022813135.1	P. decumbens FKII-L3-CM-DRAB1	GCA_022813135.1_ASM2281313v1
GenBank	GCA_002072245.1	P. decumbens IBT 11843	GCA_002072245.1_ASM207224v1
GenBank	GCA_012295545.2	P. digitatum DSM 62840	GCA_012295545.2_ASM1229554v2
GenBank	GCA_027569805.1	P. digitatum NRRL 1202	GCA_027569805.1_ASM2756980v1
GenBank	GCA_000485865.1	P. digitatum Pd01-ZJU	GCA_000485865.1_Pendig1.0
GenBank	GCA_000315645.2	P. digitatum Pd1	GCA_000315645.2_PdigPd1_v1
GenBank	GCA_001307865.1	P. digitatum PDC 102	GCA_001307865.1_ASM130786v1
GenBank	GCA_000315665.1	P. digitatum PHI26	GCA_000315665.1_PdigPHI26_v1
GenBank	GCA_015585975.1	P. dipodomyicola IF7SW-F3	GCA_015585975.1_ASM1558597v1
GenBank	GCA_015585765.1	P. dipodomyicola IIF7SW-F4	GCA_015585765.1_ASM1558576v1
GenBank	GCA_044231815.1	P. expansum 21	GCA_044231815.1_ASM4423181v1
GenBank	GCA_000769755.1	P. expansum CMP-1	GCA_000769755.1_PEX1
GenBank	GCA_000769735.1	P. expansum d1	GCA_000769735.1_ASM76973v1

Dataset	Accession no.	Name	Assembly name
GenBank	GCA_028827855.1	P. expansum IBT 35385	GCA_028827855.1_ASM2882785v1
GenBank	GCA_022829055.1	P. expansum IBT34672	GCA_022829055.1_DTU_Pex_IBT34672
GenBank	GCA_000584915.1	P. expansum NRRL 62431	GCA_000584915.1_PenAur1.0
GenBank	GCA_023624475.1	P. expansum PF2411	GCA_023624475.1_ASM2362447v1
GenBank	GCA_042477485.1	P. expansum PPRI25879	GCA_042477485.1_ASM4247748v1
GenBank	GCA_000688875.1	P. expansum R19	GCA_000688875.1_Peniclillium_expansum_v1
GenBank	GCA_004302965.1	P. expansum R19	GCA_004302965.1_Pexp_R19
GenBank	GCA_001750045.2	P. expansum R21	GCA_001750045.2_ASM175004v2
GenBank	GCA_001008385.1	P. expansum T01	GCA_001008385.1_ASM100838v1
GenBank	GCA_001513925.1	P. freii DAOM 242723	GCA_001513925.1_ASM151392v1
GenBank	GCA_037042235.1	P. freii S90-01	GCA_037042235.1_ASM3704223v1
GenBank	GCA_028827865.1	P. frequentans IBT 35679	GCA_028827865.1_ASM2882786v1
GenBank	GCA_022813365.1	P. fuscoglaucum FJII-L5-SW-P3	GCA_022813365.1_ASM2281336v1
GenBank	GCA_000576735.1	P. fuscoglaucum FM041	GCA_000576735.1_PFUSFM041_20131217
GenBank	GCA_019804545.1	P. fuscoglaucum S/N-202-OC-P2	GCA_019804545.1_ASM1980454v1
GenBank	GCA_019804555.1	P. fuscoglaucum VS AB I KN 5	GCA_019804555.1_ASM1980455v1
GenBank	GCA_047426335.1	P. griseofulvum CF3	GCA_047426335.1_ASM4742633v1
GenBank	GCA_020557365.1	P. griseofulvum D-756	GCA_020557365.1_ASM2055736v1
GenBank	GCA_001735785.1	P. griseofulvum MRI314	GCA_001735785.1_PgMRI314Ass_v1
GenBank	GCA_015585885.1	P. griseoroseum IF7SW-F5	GCA_015585885.1_ASM1558588v1
GenBank	GCA_015585865.1	P. griseoroseum IIF4SW-F4	GCA_015585865.1_ASM1558586v1
GenBank	GCA_028828265.1	P. herquei IBT 20771	GCA_028828265.1_ASM2882826v1
GenBank	GCA_028828675.1	P. herquei IBT 29812	GCA_028828675.1_ASM2882867v1
GenBank	GCA_026401425.1	P. herquei XQL_2021	GCA_026401425.1_ASM2640142v1
GenBank	GCA_001008395.1	P. italicum B3	GCA_001008395.1_PI
GenBank	GCA_000769765.1	P. italicum PHI-1	GCA_000769765.1_ASM76976v1
GenBank	GCA_002369805.1	P. janthinellum NCIM1366	GCA_002369805.1_ASM236980v1
GenBank	GCA_023626475.1	P. janthinellum PG2901	GCA_023626475.1_ASM2362647v1
GenBank	GCA_023626455.1	P. janthinellum PG2902	GCA_023626455.1_ASM2362645v1
GenBank	GCA_028827875.1	P. longicatenatum IBT 33191	GCA_028827875.1_ASM2882787v1
GenBank	GCA_028827775.1	P. malachiteum IBT 13176	GCA_028827775.1_ASM2882777v1
GenBank	GCA_028828335.1	P. malachiteum IBT 17514	GCA_028828335.1_ASM2882833v1
GenBank	GCA_902713545.1	P. manginii YELL	GCA_902713545.1_Penicillium_manginii_YELL_v2
GenBank	GCA_911174885.1	P. nalgiovense CBS112438	GCA_911174885.1_Pnal_CBS112438
GenBank	GCA_911175315.1	P. nalgiovense CBS297.97	GCA_911175315.1_Pnal_CBS297.97
GenBank	GCA_911175225.1	P. nalgiovense CBS318.92	GCA_911175225.1_Pnal_CBS318.92
GenBank	GCA_911175295.1	P. nalgiovense DTO053-A9	GCA_911175295.1_Pnal_DTO053-A9
GenBank	GCA_911174875.1	P. nalgiovense DTO204-F1	GCA_911174875.1_Pnal_DTO204-F1
GenBank	GCA_911174985.1	P. nalgiovense DTO259-D5	GCA_911174985.1_Pnal_DTO259-D5
GenBank	GCA_911175025.1	P. nalgiovense DTO334-B7	GCA_911175025.1_Pnal_DTO334-B7
GenBank	GCA_911175015.1	P. nalgiovense DTO334-C1	GCA_911175015.1_Pnal_DTO334-C1
GenBank	GCA_911174865.1	P. nalgiovense DTO335-D4	GCA_911174865.1_Pnal_DTO335-D4
GenBank	GCA_911174935.1	P. nalgiovense ESE00253	GCA_911174935.1_Pnal_ESE00253
GenBank	GCA_911175245.1	P. nalgiovense ESE00254	GCA_911175245.1_Pnal_ESE00254
GenBank	GCA_911175155.1	P. nalgiovense ESE00255	GCA_911175155.1_Pnal_ESE00255
GenBank	GCA_911175325.1	P. nalgiovense ESE00256	GCA_911175325.1_Pnal_ESE00256
GenBank	GCA_911174915.1	P. nalgiovense ESE00257	GCA_911174915.1_Pnal_ESE00257
GenBank	GCA_911174845.1	P. nalgiovense ESE00258	GCA_911174845.1_Pnal_ESE00258
GenBank	GCA_911174975.1	P. nalgiovense ESE00259	GCA_911174975.1_Pnal_ESE00259
GenBank	GCA_911174895.1	P. nalgiovense ESE00260	GCA_911174895.1_Pnal_ESE00260
GenBank	GCA_911175145.1	P. nalgiovense ESE00261	GCA_911175145.1_Pnal_ESE00261
GenBank	GCA_911175235.1	P. nalgiovense ESE00262	GCA_911175235.1_Pnal_ESE00262
GenBank	GCA_911175185.1	P. nalgiovense ESE00263	GCA_911175185.1_Pnal_ESE00263
GenBank	GCA_911175125.1	P. nalgiovense ESE00264	GCA_911175125.1_Pnal_ESE00264
GenBank	GCA_911175205.1	P. nalgiovense ESE00265	GCA_911175205.1_Pnal_ESE00265
GenBank	GCA_911175165.1	P. nalgiovense ESE00267	GCA_911175165.1_Pnal_ESE00267
GenBank	GCA_911175035.1	P. nalgiovense ESE00268	GCA_911175035.1_Pnal_ESE00268
GenBank	GCA_000577395.2	P. nalgiovense FM193	GCA_000577395.2_PNALFM193_20131217
GenBank	GCA_002072425.1	P. nalgiovense IBT 13039	GCA_002072425.1_ASM207242v1
GenBank	GCA_920106075.1	P. nalgiovense LCP03435	GCA_920106075.1_Pnal_LCP03435
GenBank	GCA_911175305.1	P. nalgiovense LCP03915	GCA_911175305.1_Pnal_LCP03915
GenBank	GCA_911639145.1	P. nalgiovense LCP03991	GCA_911639145.1_Pnal_LCP03991
GenBank	GCA_911639135.1	P. nalgiovense LCP05232	GCA_911639135.1_Pnal_LCP05232
GenBank	GCA_001278595.1	P. nordicum DAOMC 185683	GCA_001278595.1_ASM127859v1
GenBank	GCA_002382835.1	P. occitanis (nom. inval.) CT1	GCA_002382835.1_ASM238283v1
GenBank	GCA_047150385.1	P. ochrochloron hvef18	GCA_047150385.1_Poch_1.0
GenBank	GCA_911150065.1	P. olsonii CBS266.97	GCA_911150065.1_Pols_CBS266.97
GenBank	GCA_911150075.1	P. olsonii CBS626.72	GCA_911150075.1_Pols_CBS626.72
GenBank	GCA_911174955.1	P. olsonii ESE00149	GCA_911174955.1_Pols_ESE00149
GenBank	GCA_911174945.1	P. olsonii LCP04863	GCA_911174945.1_Pols_LCP04863

Dataset	Accession no.	Name	Assembly name
GenBank	GCA_025502625.1	P. olsonii WHG5	GCA_025502625.1_ASM2550262v1
GenBank	GCA_000346795.1	P. oxalicum 114-2	GCA_000346795.1_pde_v1.0
GenBank	GCA_030378375.1	P. oxalicum 5-18	GCA_030378375.1_ASM3037837v1
GenBank	GCA_021133555.1	P. oxalicum CCTCC M 20211309	GCA_021133555.1_ASM2113355v1
GenBank	GCA_022570495.1	P. oxalicum I1R1	GCA_022570495.1_ASM2257049v1
GenBank	GCA_000383025.1	P. oxalicum JU-A10-T	GCA_000383025.1_pdt_v1.0
GenBank	GCA_037043875.1	P. oxalicum LH_A471	GCA_037043875.1_ASM3704387v1
GenBank	GCA_023624855.1	P. oxalicum M7025A	GCA_023624855.1_ASM2362485v1
GenBank	GCA_023624835.1	P. oxalicum PM4501B	GCA_023624835.1_ASM2362483v1
GenBank	GCA_005546515.1	P. oxalicum SGAir0226	GCA_005546515.1_SGAir0226
GenBank	GCA_004153425.1	P. oxalicum SYJ-1	GCA_004153425.1_ASM415342v1
GenBank	GCA_004521935.1	P. oxalicum SYJ-1	GCA_004521935.1_ASM452193v1
GenBank	GCA_002072455.1	P. oxalicum YT02	GCA_002072455.1_Pexp1.0
GenBank	GCA_019191055.1	P. palitans F6_4S_1A_F	GCA_019191055.1_ASM1919105v1
GenBank	GCA_019191035.1	P. palitans F6_4S_1B_F	GCA_019191035.1_ASM1919103v1
GenBank	GCA_019191045.1	P. palitans F6_4S_1C_F	GCA_019191045.1_ASM1919104v1
GenBank	GCA_019190725.1	P. palitans F6_6S_1_F	GCA_019190725.1_ASM1919072v1
GenBank	GCA_019190485.1	P. palitans F6_7S_1A_F	GCA_019190485.1_ASM1919048v1
GenBank	GCA_019190185.1	P. palitans F6_8S_1A_F	GCA_019190185.1_ASM1919018v1
GenBank	GCA_019190165.1	P. palitans F6_8S_1C_F	GCA_019190165.1_ASM1919016v1
GenBank	GCA_019189365.1	P. palitans F8_6S_2F	GCA_019189365.1_ASM1918936v1
GenBank	GCA_019189355.1	P. palitans F8_6S_5F	GCA_019189355.1_ASM1918935v1
GenBank	GCA_019189265.1	P. palitans F8_6S-1F	GCA_019189265.1_ASM1918926v1
GenBank	GCA_019189285.1	P. palitans F8_6S-3F	GCA_019189285.1_ASM1918928v1
GenBank	GCA_019189255.1	P. palitans F8_6S-4F	GCA_019189255.1_ASM1918925v1
GenBank	GCA_902713495.1	P. palitans SP3	GCA_902713495.1_Ppalitans_SP3_v2
GenBank	GCA_031761475.1	P. pancosmium MUM 23.27	GCA_031761475.1_ASM3176147v1
GenBank	GCA_000577715.1	P. paneum FM227	GCA_000577715.1_PPANFM227_20131217
GenBank	GCA_023624535.1	P. paneum M1705	GCA_023624535.1_ASM2362453v1
GenBank	GCA_023624615.1	P. paneum PF1704S	GCA_023624615.1_ASM2362461v1
GenBank	GCA_023624515.1	P. paneum PH1708	GCA_023624515.1_ASM2362451v1
GenBank	GCA_023624635.1	P. paneum PT1701	GCA_023624635.1_ASM2362463v1
GenBank	GCA_023624575.1	P. paneum S1708	GCA_023624575.1_ASM2362457v1
GenBank	GCA_023624555.1	P. paneum Y1702	GCA_023624555.1_ASM2362455v1
GenBank	GCA_023624495.1	P. paneum Y6003	GCA_023624495.1_ASM2362449v1
GenBank	GCA_025590805.1	P. polonicum CGMCC 3.15264	GCA_025590805.1_ASM2559080v1
GenBank	GCA_025589915.1	P. polonicum CGMCC 3.15272	GCA_025589915.1_ASM2558991v1
GenBank	GCA_013466175.1	P. polonicum F7	GCA_013466175.1_ASM1346617v1
GenBank	GCA_003344595.1	P. polonicum hy4	GCA_003344595.1_ASM334459v1
GenBank	GCA_002072265.1	P. polonicum IBT 4502	GCA_002072265.1_ASM207226v1
GenBank	GCA_015585905.1	P. polonicum IIF1SW-F3	GCA_015585905.1_ASM1558590v1
GenBank	GCA_027569845.1	P. polonicum NRRL 995	GCA_027569845.1_ASM2756984v1
GenBank	GCA_025768265.1	P. polonicum PB2502	GCA_025768265.1_ASM2576826v1
GenBank	GCA_025768295.1	P. polonicum PM3203	GCA_025768295.1_ASM2576829v1
GenBank	GCA_025768855.1	P. polonicum R2416	GCA_025768855.1_ASM2576885v1
GenBank	GCA_019804565.1	P. robsamsonii VS III D KN3.2	GCA_019804565.1_ASM1980456v1
GenBank	GCA_023141385.1	P. roqueforti CBS 147308	GCA_023141385.1_ASM2314138v1
GenBank	GCA_023141315.1	P. roqueforti CBS 147309	GCA_023141315.1_ASM2314131v1
GenBank	GCA_023141305.1	P. roqueforti CBS 147310	GCA_023141305.1_ASM2314130v1
GenBank	GCA_023141205.1	P. roqueforti CBS 147311	GCA_023141205.1_ASM2314120v1
GenBank	GCA_023138675.1	P. roqueforti CBS 147317	GCA_023138675.1_ASM2313867v1
GenBank	GCA_023065495.1	P. roqueforti CBS 147318	GCA_023065495.1_ASM2306549v1
GenBank	GCA_023138645.1	P. roqueforti CBS 147325	GCA_023138645.1_ASM2313864v1
GenBank	GCA_023138615.1	P. roqueforti CBS 147326	GCA_023138615.1_ASM2313861v1
GenBank	GCA_023138445.1	P. roqueforti CBS 147330	GCA_023138445.1_ASM2313844v1
GenBank	GCA_023138355.1	P. roqueforti CBS 147331	GCA_023138355.1_ASM2313835v1
GenBank	GCA_023065595.1	P. roqueforti CBS 147332	GCA_023065595.1_ASM2306559v1
GenBank	GCA_023138015.1	P. roqueforti CBS 147333	GCA_023138015.1_ASM2313801v1
GenBank	GCA_022829115.1	P. roqueforti CBS 147337	GCA_022829115.1_ASM2282911v1
GenBank	GCA_023138005.1	P. roqueforti CBS 147338	GCA_023138005.1_ASM2313800v1
GenBank	GCA_023137995.1	P. roqueforti CBS 147339	GCA_023137995.1_ASM2313799v1
GenBank	GCA_023065465.1	P. roqueforti CBS 147354	GCA_023065465.1_ASM2306546v1
GenBank	GCA_023065415.1	P. roqueforti CBS 147355	GCA_023065415.1_ASM2306541v1
GenBank	GCA_023091085.1	P. roqueforti CBS 147372	GCA_023091085.1_ASM2309108v1
GenBank	GCA_001939915.1	P. roqueforti CECT 2905	GCA_001939915.1_ASM193991v1
GenBank	GCA_023141515.1	P. roqueforti DTO 002-I6	GCA_023141515.1_ASM2314151v1
GenBank	GCA_023141395.1	P. roqueforti DTO 003-C3	GCA_023141395.1_ASM2314139v1
GenBank	GCA_023065655.1	P. roqueforti DTO 006-G1	GCA_023065655.1_ASM2306565v1
GenBank	GCA_023141355.1	P. roqueforti DTO 006-G7	GCA_023141355.1_ASM2314135v1
GenBank	GCA_023065685.1	P. roqueforti DTO 012-A1	GCA_023065685.1_ASM2306568v1

Dataset	Accession no.	Name	Assembly name
GenBank	GCA_023141275.1	P. roqueforti DTO 012-A7	GCA_023141275.1_ASM2314127v1
GenBank	GCA_023065625.1	P. roqueforti DTO 012-A8	GCA_023065625.1_ASM2306562v1
GenBank	GCA_023141235.1	P. roqueforti DTO 012-A9	GCA_023141235.1_ASM2314123v1
GenBank	GCA_023141195.1	P. roqueforti DTO 013-E5	GCA_023141195.1_ASM2314119v1
GenBank	GCA_023065525.1	P. roqueforti DTO 013-F2	GCA_023065525.1_ASM2306552v1
GenBank	GCA_023141165.1	P. roqueforti DTO 027-I6	GCA_023141165.1_ASM2314116v1
GenBank	GCA_023141115.1	P. roqueforti DTO 032-C6	GCA_023141115.1_ASM2314111v1
GenBank	GCA_023141145.1	P. roqueforti DTO 039-G3	GCA_023141145.1_ASM2314114v1
GenBank	GCA_023141155.1	P. roqueforti DTO 046-C5	GCA_023141155.1_ASM2314115v1
GenBank	GCA_000513255.1	P. roqueforti FM164	GCA_000513255.1_PROQFM164_20130607
GenBank	GCA_001599855.1	P. roqueforti JCM 22842	GCA_001599855.1_JCM_22842_assembly_v001
GenBank	GCA_023065375.1	P. roqueforti LCP 96.3914a	GCA_023065375.1_ASM2306537v1
GenBank	GCA_030518555.1	P. roqueforti LCP06133	GCA_030518555.1_ASM3051855v1
GenBank	GCA_000737485.2	P. roqueforti UASWS P1	GCA_000737485.2_PrP1-1.0
GenBank	GCA_902636305.1	P. rubens	GCA_902636305.1_nPRUBv1
GenBank	GCA_027256995.2	P. rubens 212	GCA_027256995.2_ASM2725699v2
GenBank	GCA_011058885.1	P. rubens 43M1	GCA_011058885.1_ASM1105888v1
GenBank	GCA_019189275.1	P. rubens F8_6S_6F	GCA_019189275.1_ASM1918927v1
GenBank	GCA_019189245.1	P. rubens F8_6S_7F	GCA_019189245.1_ASM1918924v1
GenBank	GCA_028828505.1	P. rubens IBT 35670	GCA_028828505.1_ASM2882850v1
GenBank	GCA_037044095.1	P. rubens LH_A166	GCA_037044095.1_ASM3704409v1
GenBank	GCA_027257005.2	P. rubens S27	GCA_027257005.2_ASM2725700v2
GenBank	GCA_000226395.1	P. rubens Wisconsin 54-1255	GCA_000226395.1_PenChr_Nov2007
GenBank	GCA_028751425.1	P. rubens YAP1	GCA_028751425.1_UCR_PencilliumYAP1_1.1
GenBank	GCA_911175135.1	P. salamii CBS135391	GCA_911175135.1_Psal_CBS135391
GenBank	GCA_911175275.1	P. salamii CBS135392	GCA_911175275.1_Psal_CBS135392
GenBank	GCA_911175105.1	P. salamii CBS135393	GCA_911175105.1_Psal_CBS135393
GenBank	GCA_911174905.1	P. salamii CBS135394	GCA_911174905.1_Psal_CBS135394
GenBank	GCA_911175075.1	P. salamii CBS135395	GCA_911175075.1_Psal_CBS135395
GenBank	GCA_911174965.1	P. salamii CBS135396	GCA_911174965.1_Psal_CBS135396
GenBank	GCA_911174925.1	P. salamii CBS135397	GCA_911174925.1_Psal_CBS135397
GenBank	GCA_911175095.1	P. salamii CBS135399	GCA_911175095.1_Psal_CBS135399
GenBank	GCA_911175285.1	P. salamii CBS135400	GCA_911175285.1_Psal_CBS135400
GenBank	GCA_911639275.1	P. salamii CBS135401	GCA_911639275.1_Psal_CBS135401
GenBank	GCA_911197385.1	P. salamii CBS135403	GCA_911197385.1_Psal_CBS135403
GenBank	GCA_911197295.1	P. salamii CBS135407	GCA_911197295.1_Psal_CBS135407
GenBank	GCA_911197175.1	P. salamii CCFL11ab2.1	GCA_911197175.1_Psal_CCFL11ab2.1
GenBank	GCA_911197195.1	P. salamii CCFL5	GCA_911197195.1_Psal_CCFL5
GenBank	GCA_911197305.1	P. salamii DTO032-F3	GCA_911197305.1_Psal_DTO032-F3
GenBank	GCA_911197205.1	P. salamii DTO198-E3	GCA_911197205.1_Psal_DTO198-E3
GenBank	GCA_911197365.1	P. salamii DTO334-B6	GCA_911197365.1_Psal_DTO334-B6
GenBank	GCA_911197325.1	P. salamii LCP06521	GCA_911197325.1_Psal_LCP06521
GenBank	GCA_911197405.1	P. salamii LCP06522	GCA_911197405.1_Psal_LCP06522
GenBank	GCA_911197245.1	P. salamii LCP06523	GCA_911197245.1_Psal_LCP06523
GenBank	GCA_911456365.1	P. salamii LCP06525	GCA_911456365.1_Psal_LCP06525
GenBank	GCA_001750025.1	P. sclerotiorum 113	GCA_001750025.1_ASM175002v1
GenBank	GCA_027569435.1	P. sclerotiorum NRRL 2074	GCA_027569435.1_ASM2756943v1
GenBank	GCA_027569445.1	P. simplicissimum NRRL 1075	GCA_027569445.1_ASM2756944v1
GenBank	GCA_027569465.1	P. simplicissimum NRRL 2016	GCA_027569465.1_ASM2756946v1
GenBank	GCA_002072235.1	P. solitum IBT 29525	GCA_002072235.1_ASM207223v1
GenBank	GCA_023624715.1	P. solitum M7003A	GCA_023624715.1_ASM2362471v1
GenBank	GCA_001750005.1	P. solitum NJ1	GCA_001750005.1_ASM175000v1
GenBank	GCA_023624775.1	P. solitum PB3502S	GCA_023624775.1_ASM2362477v1
GenBank	GCA_023624755.1	P. solitum PL1801	GCA_023624755.1_ASM2362475v1
GenBank	GCA_000952775.2	P. solitum RS1	GCA_000952775.2_ASM95277v2
GenBank	GCA_023624695.1	P. solitum S2401	GCA_023624695.1_ASM2362469v1
GenBank	GCA_023624785.1	P. solitum S2705F	GCA_023624785.1_ASM2362478v1
GenBank	GCA_023624735.1	P. solitum Y2401	GCA_023624735.1_ASM2362473v1
GenBank	GCA_023624815.1	P. solitum Y2401C	GCA_023624815.1_ASM2362481v1
GenBank	GCA_039702135.1	P. sp. 32 TS-2023	GCA_039702135.1_ASM3970213v1
GenBank	GCA_030710325.1	P. sp. 91 TS-2023	GCA_030710325.1_ASM3071032v1
GenBank	GCA_037042535.1	P. sp. B98-03	GCA_037042535.1_ASM3704253v1
GenBank	GCA_029142745.1	P. sp. BM32	GCA_029142745.1_NB24_v1
GenBank	GCA_008931925.1	P. sp. BW_12	GCA_008931925.1_ASM893192v1
GenBank	GCA_008931945.1	P. sp. BW_162_3FA	GCA_008931945.1_ASM893194v1
GenBank	GCA_008931935.1	P. sp. BW_MB	GCA_008931935.1_ASM893193v1
GenBank	GCA_037356175.1	P. sp. C7(2024)	GCA_037356175.1_NIH_C7_1.1
GenBank	GCA_005250745.2	P. sp. CF01	GCA_005250745.2_ASM525074v2
GenBank	GCA_002916455.1	P. sp. CF05	GCA_002916455.1_ASM291645v1
GenBank	GCA_028827225.1	P. sp. CMV-2018d IBT 12396	GCA_028827225.1_ASM2882722v1

Dataset	Accession no.	Name	Assembly name
GenBank	GCA_025768115.1	P. sp. D2Mb R2202	GCA_025768115.1_ASM2576811v1
GenBank	GCA_037356155.1	P. sp. D9	GCA_037356155.1_NIH_D9_1.1
GenBank	GCA_036712075.1	P. sp. DT28	GCA_036712075.1_ASM3671207v1
GenBank	GCA_028827535.1	P. sp. DV-2018c IBT 19332	GCA_028827535.1_ASM2882753v1
GenBank	GCA_028827425.1	P. sp. DV-2018c IBT 6001	GCA_028827425.1_ASM2882742v1
GenBank	GCA_035048945.1	P. sp. E22	GCA_035048945.1_UMS_E22
GenBank	GCA_037414295.1	P. sp. F2(2024)	GCA_037414295.1_NIH_F2_1.1
GenBank	GCA_037356195.1	P. sp. F50	GCA_037356195.1_NIH_F50_1.1
GenBank	GCA_037356495.1	P. sp. F51	GCA_037356495.1_NIH_F51_1.1
GenBank	GCA_027569595.1	P. sp. G339	GCA_027569595.1_ASM2756959v1
GenBank	GCA_027569895.1	P. sp. G342	GCA_027569895.1_ASM2756989v1
GenBank	GCA_049463015.1	P. sp. GS218	GCA_049463015.1_ASM4946301v1
GenBank	GCA_002000375.1	P. sp. HKF2	GCA_002000375.1_ASM200037v1
GenBank	GCA_028828865.1	P. sp. IBT 16267x	GCA_028828865.1_ASM2882886v1
GenBank	GCA_028828935.1	P. sp. IBT 18751x	GCA_028828935.1_ASM2882893v1
GenBank	GCA_028827885.1	P. sp. IBT 31633x	GCA_028827885.1_ASM2882788v1
GenBank	GCA_028828895.1	P. sp. IBT 35674x	GCA_028828895.1_ASM2882889v1
GenBank	GCA_047663515.1	P. sp. INA 01369	GCA_047663515.1_ASM4766351v1
GenBank	GCA_037042505.1	P. sp. M121-12C	GCA_037042505.1_ASM3704250v1
GenBank	GCA_023626435.1	P. sp. M1327 PF1803	GCA_023626435.1_ASM2362643v1
GenBank	GCA_003138045.1	P. sp. MA 6036	GCA_003138045.1_ASM313804v1
GenBank	GCA_003138025.1	P. sp. MA 6040	GCA_003138025.1_ASM313802v1
GenBank	GCA_030779445.1	P. sp. MBC 424	GCA_030779445.1_ASM3077944v1
GenBank	GCA_030779285.1	P. sp. MBC 428	GCA_030779285.1_ASM3077928v1
GenBank	GCA_003852855.1	P. sp. MT2 MMC-2018	GCA_003852855.1_ASM385285v1
GenBank	GCA_029142755.1	P. sp. MT45	GCA_029142755.1_NB21_v1
GenBank	GCA_037074905.1	P. sp. MYA5	GCA_037074905.1_ASM3707490v1
GenBank	GCA_011750695.1	P. sp. OUCMDZ-019	GCA_011750695.1_ASM1175069v1
GenBank	GCA_023626575.1	P. sp. PF1803 PT4103	GCA_023626575.1_ASM2362657v1
GenBank	GCA_023626555.1	P. sp. PF2412A R1202D	GCA_023626555.1_ASM2362655v1
GenBank	GCA_037042255.1	P. sp. PG106-07D	GCA_037042255.1_ASM3704225v1
GenBank	GCA_037042135.1	P. sp. PG113-03A	GCA_037042135.1_ASM3704213v1
GenBank	GCA_037042625.1	P. sp. PG115-01	GCA_037042625.1_ASM3704262v1
GenBank	GCA_023626615.1	P. sp. PH3801 S1316	GCA_023626615.1_ASM2362661v1
GenBank	GCA_023626495.1	P. sp. PT4103 D2Mb	GCA_023626495.1_ASM2362649v1
GenBank	GCA_023626515.1	P. sp. R1202D S1126A	GCA_023626515.1_ASM2362651v1
GenBank	GCA_037042055.1	P. sp. S103-07	GCA_037042055.1_ASM3704205v1
GenBank	GCA_023626535.1	P. sp. S1126A PH3801	GCA_023626535.1_ASM2362653v1
GenBank	GCA_023627405.1	P. sp. S1316 R2504	GCA_023627405.1_ASM2362740v1
GenBank	GCA_023626355.1	P. sp. S1602 S1701	GCA_023626355.1_ASM2362635v1
GenBank	GCA_023626415.1	P. sp. S1701 PF2412A	GCA_023626415.1_ASM2362641v1
GenBank	GCA_003800495.2	P. sp. SPG-F1	GCA_003800495.2_ASM380049v2
GenBank	GCA_003800485.2	P. sp. SPG-F15	GCA_003800485.2_ASM380048v2
GenBank	GCA_013138035.1	P. sp. str. #12	GCA_013138035.1_ASM1313803v1
GenBank	GCA_019775275.1	P. sp. VS AB III KN	GCA_019775275.1_ASM1977527v1
GenBank	GCA_019775305.1	P. sp. VS AB III KN 1	GCA_019775305.1_ASM1977530v1
GenBank	GCA_019775415.1	P. sp. VS I D KN	GCA_019775415.1_ASM1977541v1
GenBank	GCA_019775465.1	P. sp. VS I D KN 3	GCA_019775465.1_ASM1977546v1
GenBank	GCA_004959885.1	P. sp. W3 MMC-2018	GCA_004959885.1_SRS-W-3
GenBank	GCA_029142805.1	P. sp. WT45	GCA_029142805.1_NB22_v1
GenBank	GCA_002072375.1	P. steckii IBT 24891	GCA_002072375.1_ASM207237v1
GenBank	GCA_001908125.1	P. subrubescens CBS 132785	GCA_001908125.1_ASM190812v1
GenBank	GCA_022813665.1	P. sumatraense FJI-L10-BK-P2	GCA_022813665.1_ASM2281366v1
GenBank	GCA_022813645.1	P. sumatraense FJI-L3-BK-DG1	GCA_022813645.1_ASM2281364v1
GenBank	GCA_037041715.1	P. sumatraense R121-14B	GCA_037041715.1_ASM3704171v1
GenBank	GCA_028751415.1	P. turbatum blh34	GCA_028751415.1_ASM2875141v1
GenBank	GCA_014839855.1	P. ucsense 2HH	GCA_014839855.1_UCS_PECH_1.0
GenBank	GCA_028828565.1	P. verhagenii IBT 33313	GCA_028828565.1_ASM2882856v1
GenBank	GCA_000970515.2	P. verrucosum BFE808	GCA_000970515.2_PV808_April18ReSeqReAss
GenBank	GCA_025585045.1	P. verrucosum IFST Pver1	GCA_025585045.1_ASM2558504v1
GenBank	GCA_025585095.1	P. verrucosum IFST Pver2	GCA_025585095.1_ASM2558509v1
GenBank	GCA_902713535.1	P. viridicatum XA	GCA_902713535.1_Penicillium_viridicatum_XA_v2
GenBank	GCA_002072255.1	P. vulpinum IBT 29486	GCA_002072255.1_ASM207225v1
GenBank	GCA_027569495.1	P. vulpinum NRRL 1001	GCA_027569495.1_ASM2756949v1
GenBank	GCA_001305275.1	Talaromyces verruculosus TS63-9	GCA_001305275.1_ASM130527v1

Supplementary Table S3: DNA barcode analysis. Results from alignment of the 65 main cluster isolate barcodes against reference barcodes from the *Penicillium* section *Fasciculata* series *Camembertiorum* species, plus *P. fuscoglaucum*.

	DNA barcodes (ITS, BenA, CaM, RPB2)*	*Differences / reference length*			
		ITS	*BenA*	*CaM*	*RPB2*
P. biforme	KC411731, MN969373, KU896823, KU904346	0 / 508	5 / 459	0 / 514	4 / 1020
P. camemberti	AB479314, FJ930956, KU896825, MN969109	1 / 610	0 / 380	1 / 493	1 / 1014
P. caseifulvum	KJ834504, AY674372, KU896826, KU904347	0 / 693	4 / 428	0 / 510	1 / 1109
P. cavernicola	KJ834505, KJ834439, KU896827, KU904348	1 / 693	11 / 406	21 / 511	18 / 1165
P. commune	AY213672, MN969377, KU896829, KU904350	0 / 586	1 / 459	0 / 510	1 / 1083
P. crustosum	AF033472, MN969379, DQ911132, MN969114	3 / 1143	14 / 456	17 / 575	18 / 1014
P. discolor	AJ004816, AY674348, KU896834, KU904351	1 / 546	20 / 479	20 / 514	21 / 1074
P. echinulatum	AF033473, AY674341, DQ911133, KU904352	2 / 1143	15 / 433	24 / 576	20 / 1104
P. fuscoglaucum	MH855062, OR206420, OR206421, OR206422	0 / 817	3 / 429	1 / 534	1 / 1053
P. palitans	KJ834514, KJ834480, KU896847, KU904360	0 / 693	7 / 385	0 / 501	10 / 1100
P. solitum	AY373932, MN969398, KU896851, KU904363	1 / 610	14 / 456	16 / 503	18 / 1054
P. speluncae	MG490869, MG490889, MG490959, MN170741	1 / 811	16 / 450	16 / 504	20 / 1093

*All barcodes were from Houbraken et al. (2020), except those from *P. fuscoglaucum*, which were from Visagie et al. (2024).

Houbraken, J., Kocsube, S., Visagie, C.M., Yilmaz, N., Wang, X.C., Meijer, M., Kraak, B., Hubka, V., Bensch, K., Samson, R.A., Frisvad, J.C. (2020). Classification of *Aspergillus*, *Penicillium*, *Talaromyces* and related genera (*Eurotiales*): An overview of families, genera, subgenera, sections, series and species. Stud. Mycol. 95, 5-169. https://doi.org/10.1016/j.simyco.2020.05.002.

Visagie, C.M., Yilmaz, N., Kocsube, S., Frisvad, J.C., Hubka, V., Samson, R.A., Houbraken, J. (2024). A review of recently introduced *Aspergillus*, *Penicillium*, *Talaromyces* and other *Eurotiales* species. Stud. Mycol. 107, 1-66. https://doi.org/10.3114/sim.2024.107.01.

Supplementary Table S4: Statistical assessment of whether specific potato suppliers or production dates were overrepresented within phylogenetic subclusters containing 5 to 30 isolates in the ML tree shown in Figure 2. Statistics are reported as uncorrected Fisher's exact test p-values and Benjamini–Hochberg FDR-adjusted p-values.

Sub-cluster id	Boot-strap	Clade size	No. of product isolates	Number of isolates from each supplier in clade	Date span days	Overrepresentation of most enriched supplier in clade			Temporal clustering in clade	
						Supplier	p	p after FDR	p	P after FDR
Result from assessment of subclusters with bootstrap support ≥95:										
C1	96	18	8	Farm A:8	208	Farm A	**0.003**	**0.010**	0.125	0.627
C2	100	9	3	Farm A:1,Farm B:1,Farm D:1	127				0.372	0.919
C3	100	5	2	Farm B:2	88				0.551	0.919
C4	99	11	7	Farm A:6,Farm B:1	369	Farm A	0.094	0.141	0.987	0.998
C5	100	14	10	Farm A:6,Farm B:2,Farm C:1,Farm D:1	440	Farm D	1	1	0.998	0.998
Result for additional clusters tested in assessment of subclusters with bootstrap support ≥85:										
C6	87	7	4	Farm A:1,Farm C:2,Farm U:1	102				0.414	0.829
C7	93	10	4	Farm A:1,Farm B:2,Farm C:1	361				0.698	0.931
C8	91	5	3	Farm A:3	200				0.337	0.829

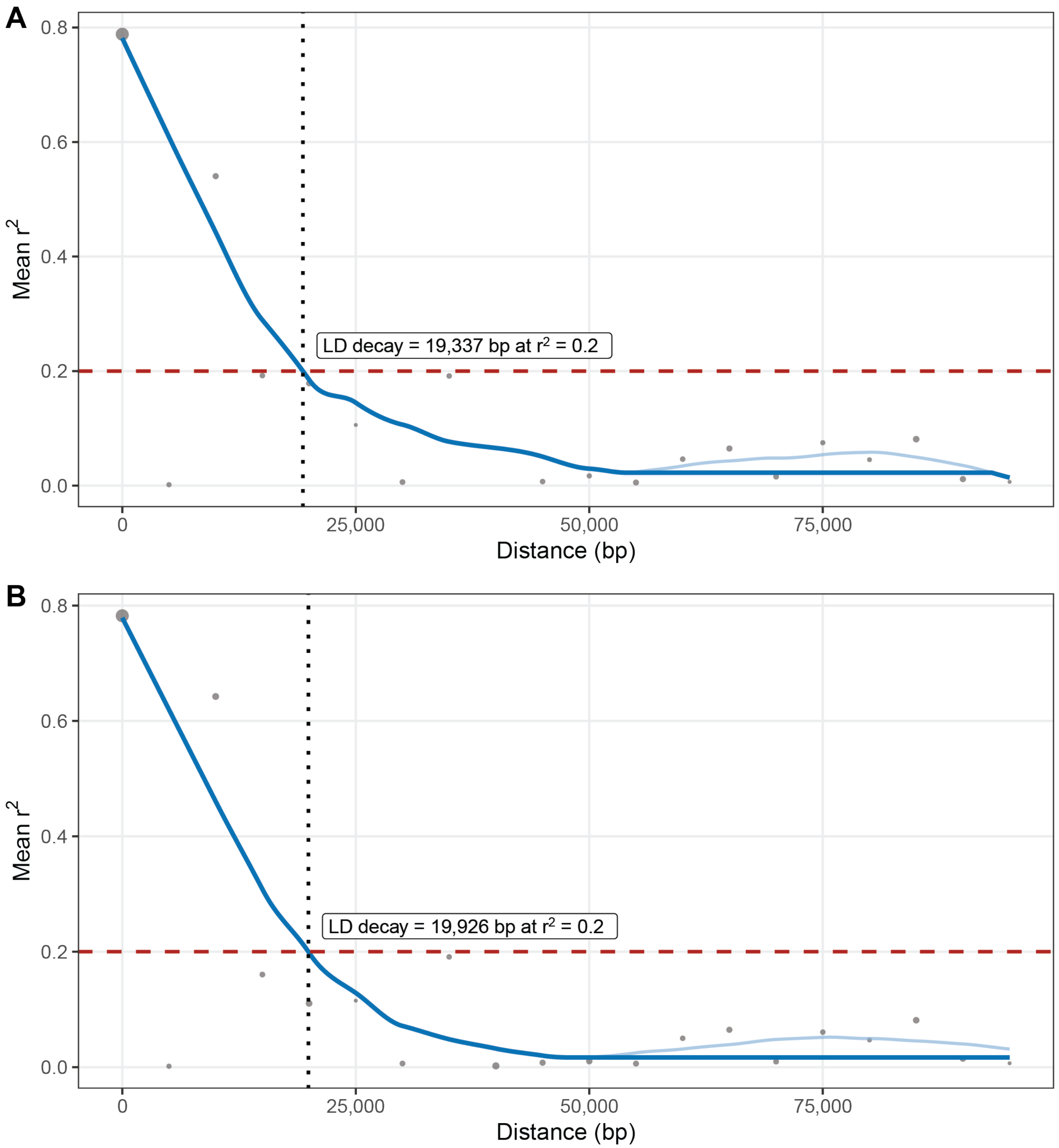

Supplementary Figure S1. Linkage disequilibrium (LD) decay in *P. commune* genomes based on pairwise SNP comparisons. LD was quantified as mean r^2 in 5 kb bins and smoothed using LOESS weighted by bin counts. Two datasets are shown: A) all 65 isolates including MF09489 and B) 64 isolates after removal of MF09489. LD decayed to $r^2 \leq 0.2$ at approximately 19-20 kb in both datasets.

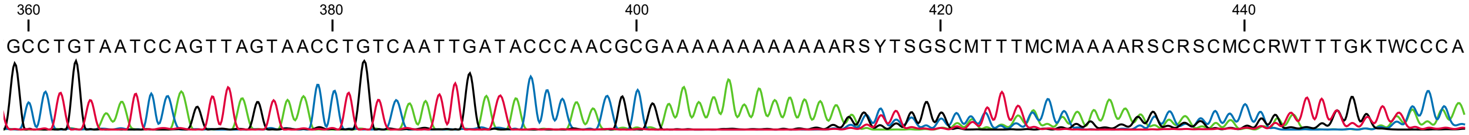

Supplementary Figure S2: Sanger sequencing chromatogram of the *BenA* region from isolate MF09504 using the reverse primer Bt2b. The poly-T tract appears as an A-tract due to sequencing orientation. Overlapping peaks after this region indicate a mixed population of PCR products differing by one base, consistent with polymerase slippage during amplification.

Supplementary Figure S3. Full Mash-based Neighbor-Joining tree of 68 bakery isolates and 622 reference genomes representing *Penicillium* genomes and *Aspergillus niger* ATCC 1015, used as the outgroup. Isolates from the current study are shown in green, genomes from Ropars *et al.* (2020) in orange, RefSeq reference genomes in red, and other GenBank genomes in black. Branch lengths are proportional to Mash distance, as indicated by the scale bar. This figure shows the complete dataset; a condensed version focusing on series *Camembertiorum* is presented in Figure 1.

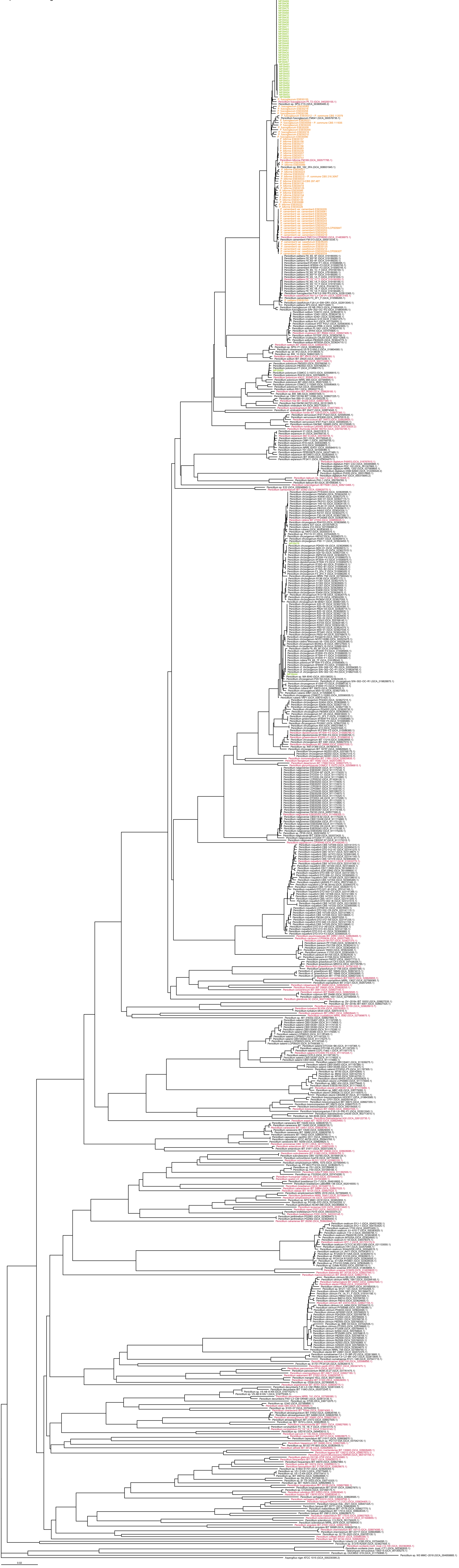

Supplement: Supplementary file 1 [file DataSheet1.pdf]
